# Per- and Polyfluoroalkyl Substances in the Duluth, Minnesota Area: Exposure to and Biomarker Responses in Tree Swallows Relative to Known Fire-Fighting Foam Sources

**DOI:** 10.3390/toxics12090660

**Published:** 2024-09-10

**Authors:** Christine M. Custer, Paul M. Dummer, Matthew A. Etterson, Jonathan T. Haselman, Sandra Schultz, Natalie Karouna-Renier, Cole Matson

**Affiliations:** 1 Upper Midwest Environmental Sciences Center, U.S. Geological Survey, 2630 Fanta Reed Road, La Crosse, WI 54603, USA; pdummer@usgs.gov; 2 U.S. EPA, Office of Research and Development, Great Lakes Toxicology and Ecology Division, 6201 Congdon Blvd, Duluth, MN 55804, USA; etterson.matthew@epa.gov (M.A.E.); haselman.jon@epa.gov (J.T.H.); 3 Eastern Ecological Science Center at Patuxent, U.S. Geological Survey, 12100 Beech Forest Rd., Laurel, MD 20708, USAnkarouna@usgs.gov (N.K.-R.); 4 Department of Environmental Science, Baylor University, Waco, TX 76798, USA; cole_matson@baylor.edu

**Keywords:** biomarker responses, Duluth, PFAS, tree swallows, perfluorohexane sulfonate

## Abstract

Tree swallow nest boxes were deployed at sites proximal to two putative aqueous film forming foam (AFFF) sources in the Duluth, MN area, as well as along the St. Louis River and a reference lake for comparative purposes in 2019, 2020 and 2021. The two AFFF sites were the current Duluth Air National Guard Base (ANG) and the Lake Superior College Emergency Response Training Center. Between 13 and 40 per- and polyfluoroalkyl substances (PFAS), depending on year, were detected and quantified in tree swallow egg, nestling carcasses, and stomach contents. Assessments were made of oxidative stress and ethoxyresorufin-O-dealkylase activity in liver tissue, thyroid hormone levels in plasma and thyroid glands, DNA damage in red blood cells, and two measures of immune response (haptoglobin-like activity and immunoglobulin) in plasma of the nestlings. Additionally, other contaminants, such as polychlorinated biphenyls, legacy organochlorine pesticides, and trace elements, were assessed at sites with no previous data. Total egg PFAS concentrations at the ANG site and north of that site were 30–40 times higher than at the reference lake, while nestling PFAS concentrations were 10–15 times higher. In contrast, the St. Louis River sites had slightly, but non-statistically significant, elevated egg and nestling PFAS concentrations relative to the reference lake (2–5 times higher). One PFAS, perfluorohexane sulfonate (PFHxS), was higher, as a proportion of total PFAS, at sites with a known AFFF source compared to the reference lake, as well as compared to sites along the St. Louis River with mainly urban and industrial sources of PFAS. The ratio of total carboxylates to total sulfonates also distinguished between PFAS sources. There were few to no differences in biomarker responses among sites, and no association with PFAS exposure.

## 1. Introduction

Per- and polyfluoroalkyl substances (PFAS) are a global contaminant. Refer to the many reviews, such as [1,2,3], for history, uses, and ecotoxicological information, and Fenton et al. [4] and Fromme et al. [5] for human exposure review. Additionally, PFAS were first recognized as having worldwide distribution in birds by Giesy and Kannan [6] with the sources for those exposures being both varied and numerous. Many PFAS sources are non-point, also referred to as indirect sources, (after terminology in [3], which include atmospheric deposition and sources from consumer products (food packaging, non-stick cookware, stain- and water- repellent products), or those used in industrial processes (Gaines [7] for a review) that often manifest themselves at wastewater treatment plant (WWT) outfalls. Although WWT plants are considered point sources in a regulatory context, we have classified them as an indirect source because the incoming water is received from many different, and often unknown, PFAS sources, the point being that the PFAS did not originate at the WWT plant. Point sources, for the purposes of this paper, include PFAS manufacturing plants, but also locations where fire suppressant foams were known to be used or where training to use these products was performed. These foams were used at both military and commercial airports. While training to use these foams has occurred primarily at military bases, there are also training programs at colleges and technical schools that teach fire management skills.

The Duluth, MN area was an ideal location to study PFAS exposure as well as potential effects because the Duluth area was found to be one of two sites with greatly elevated PFAS in tree swallow plasma samples in a comprehensive study across all five Great Lakes [8]. Additionally, in the same geographical area, there were also locations with more modest and even background exposure to PFAS, which allowed for comparisons among potentially different source types. Having different exposure levels within the same region is especially important when analyzing biomarker responses, which could possibly be influenced by climatological or other natural factors intrinsic to a region.

Tree swallows are widely used as a model study species for ecotoxicological research [9] because they can be attracted to specific areas of interest and their diet closely reflects sediment contamination. Over the years, a suite of physiological biomarkers has been developed for use with tree swallows [10,11]. Many of these biomarkers are responsive to exposure to legacy contaminants, such as polychlorinated biphenyls (PCBs), polycyclic aromatic hydrocarbons, dioxins and furans, i.e., those acting on the aryl hydrocarbon receptor pathway, but can also be affected by some trace elements; it is unknown if these biomarkers may also be used to access adverse responses from exposure to PFAS chemicals. Two immune response biomarkers were assessed because there is evidence from other wildlife species that immune function can be compromised because of PFAS exposure [2]. The objectives of this study were to quantify PFAS and other contaminant exposures in tree swallow tissues and their diet in the St. Louis River, MN, as well as in the greater Duluth, MN areas, to understand the geographic distribution of these contaminant classes, to assess whether there was an association with PFAS and this suite of bio-marker responses, and finally to explore methods to distinguish PFAS sources in avian tissues.

## 2. Materials and Methods

**Figure 1:**
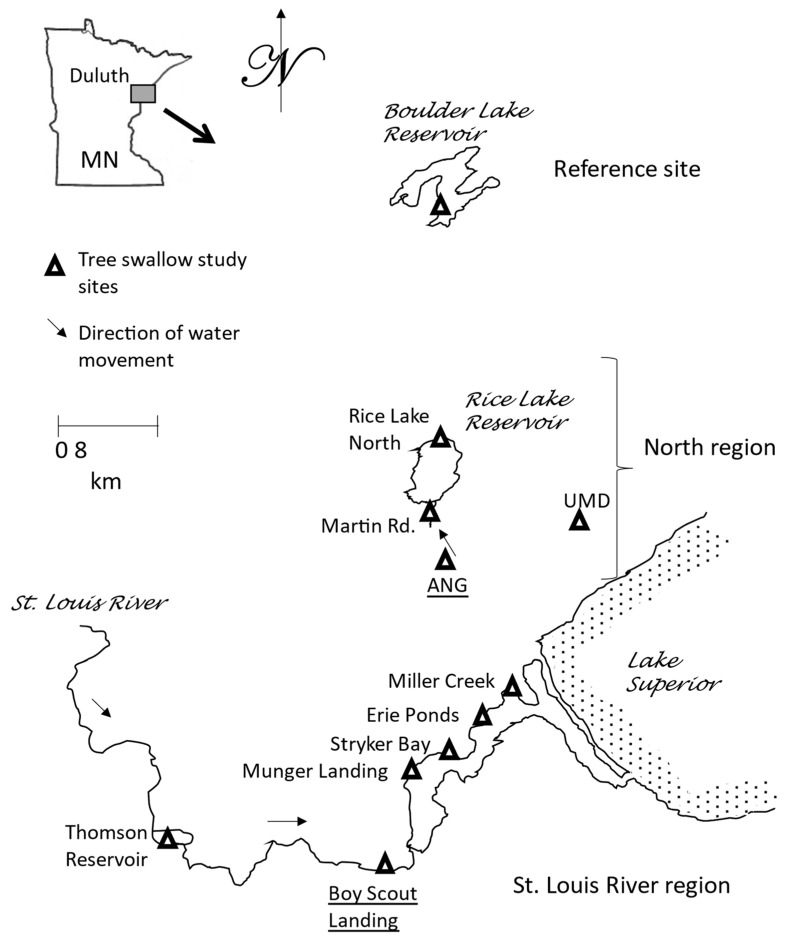
Map of per- and polyfluorinated substances (PFAS) study sites in the Duluth, MN area 2019–2021. Two underlined site names are known as locations at which aqueous film forming foams (AFFFs) were used. Note: ANG = Duluth Air National Guard Base and UMD = farm at University of Minnesota, Duluth.

Tree swallow nest boxes (n = 15 to 20 per site) were either already deployed or were newly erected in 2019, 2020 and 2021 at sites in the Duluth, MN area (Figure 1).

Nest boxes were attached to metal posts and protected from ground predators using cylindrical metal vent pipes. Tree swallows that breed across the northern tier of states in the U.S. [12], as well as into Canada, are cavity limited, so they will readily find and occupy man-made nest boxes. These methods have been used to study not only the natural history of this species, but also contaminant exposure and effects (for a review, refer to [9]. Boxes were deployed at sites proximal to two putative aqueous film forming fire-fighting foam (AFFF) sources, the current Duluth Air National Guard Base (ANG) and the Lake Superior College Emergency Response Training Center (ERTC) near Boy Scout Landing on the St. Louis River. Usage of AFFF at the ANG site in Duluth included fire training areas, as well as the fire station and hangers; the fire training areas were actively used between 1951 and 1987 on a twice/month schedule [13]. There were no emergency or crash sites at ANG where AFFF was used. Lake Superior College began their training classes in 1994 and has trained over 5000 students since them (https://www.lsc.edu/safety/pfas/, accessed on 8 February 2024). Five sites were sampled along the St. Louis River, one upstream from Boy Scout Landing and four downstream. Two sites were north (down gradient hydrologically) of the ANG site. Finally, a reference site, Boulder Lake, was established, as well as a more terrestrial location on University of Minnesota, Duluth (UMD) property, where PFAS exposure was also expected to be minimal, to document background conditions and to provide a reference site for the biomarker analyses.

Beginning at egg laying (mid- to late May), nest boxes were monitored weekly until nestlings reached 12 days of age, generally in late June or early July. Egg and nestling samples were collected from up to 10 nest boxes per site for chemical exposure assessment. Egg samples (n = 2 eggs per nest box) were collected from nest boxes shortly after egg-laying was completed. Nestlings (n = 1 or 2 per nest box) were collected from the same nest boxes, when possible, at 12-days of age (±1 day of age). More details on field methods are in Custer et al. ([14], for eggs) and in Custer et al. ([8], for nestlings). After nestlings were collected, erupted feather length of the first primary was measured; they were weighed on a pan balance (0.01 g), euthanized using approved AMVA [15] methods, and dissected appropriately for the specific analyses, i.e., stomach contents (hereafter ‘diet’), liver, blood, two thyroid glands, and whole carcass remainder. Diet samples were removed from the stomachs of each nestling and ultimately composited by site because of sample mass requirements for analytical chemistry. Each constituent for chemical analysis was placed in certified chemically clean jars (Thermo Fisher Scientific, Waltham, MA USA) and frozen in a standard freezer (−20 °C) until analyzed. Aliquots of liver tissue for biomarker assessments were dissected and immediately snap frozen in a vapor-phase nitrogen tank and held there until transferred to a −80 °C freezer. Blood was collected in heparinized tubes and centrifuged to separate the plasma from the red blood cells. The plasma samples were kept frozen in a standard freezer, while the red blood cells were first mixed with Hamm’s freezing media and then immediately frozen in the vapor-phase nitrogen tank as above. Animal handling procedures were reviewed and approved by U. S. Geological Survey’s Upper Midwest Environmental Sciences Center Animal Care and Use Committee, and all collections were made under applicable Federal, State, and local permits.

### 2.1. Chemical Analyses

Egg samples and nestling carcasses were analyzed individually for 13 PFAS (2019), 33 PFAS (2020), and 40 PFAS (2021, Supplementary Table S1). The difference in number of analytes analyzed each year reflected the current number of commercially available PFAS analytes. While PFAS were the primary focus of this study, a small subset of egg and nestling samples were analyzed for PCBs, legacy organochlorine pesticides (n = 35), PBDEs, and n = 22 trace elements to augment previously collected data [8,14]. These data will help inform the potential contribution of co-occurring contaminants to the measured effect endpoints. Total toxic equivalency (TEQ) for PCBs was also calculated using the World Health Organization’s toxic equivalent factors [16], which was done by multiplying the individual concentrations by the factor provided in that publication and summing them. Chemical analyses were performed by SGS AXYS Analytical Services Ltd., Sidney, British Columbia, Canada. Chemical methodologies followed standard EPA methods for 13 individual PFAS (2019 and 2020) and for the additional 27 PFAS in 2021 [17]. Standard methods were also used for 209 PCB congeners, 209 PBDE congeners, and legacy insecticides [18,19,20]. More details can be found in Custer et al. [8,14,21] but, in brief, samples were homogenized, extracted, and cleaned using columns appropriate for the chemical class being analyzed. Analyses were by gas chromatography/mass spectrometry (GS/MS) for PBDEs, PCBs and organochlorine chemicals, or by liquid chromatography/mass spectrometry (LC/MS) for PFAS. Concentrations of specific PFAS were determined by isotope dilution or internal standard quantification. Blanks, spikes and duplicates were analyzed with each batch (~20 samples per batch); certified reference material was also analyzed. Concentrations are reported as ng/g wet wt. for all PFAS and organochlorine contaminations, PBDEs are expressed as pg/g wet wt., and trace elements as µg/g dry wt.

Although there were some differences in detection limits (DLs) for PFAS by year and matrix (Supplementary Table S1 for those specifics), they were generally consistent. In brief, average DLs for individual PFAS were generally <0.25 ng/g wet wt. for both eggs and nestlings for most PFAS, except as noted below. Carboxylic acids and sulfonates had slightly higher DLs, between 0.26 and 0.50 ng/g, in 2019 nestlings. Detection limits were between 0.26 and 1.50 for perfluorobutanoate (PFBA), but also for the fluorotelomer sulfonates, sulfonamides, and ether carboxylates and sulfonates. Detection limits for the fluorotelomer carboxylates were between 2.5 and 4.0 ng/g. Overall DLs for diet samples were similar as those for eggs and nestlings. Average percent recovery for PFAS was 104% and ranged from 59.2% (PFDoS) to 185% (N-MeFOSE, Supplementary Table S2). Recoveries for 83% of PFAS were between 80 and 120%. Concentrations of total PFAS_13_, PFAS_30_, and PFAS_40_ were the sum of all PFAS, either 13, 30, or 40 PFAS, respectively, which had concentrations > the DL. PFAS_13_ enables comparison with earlier data, including the 2019 nestling data that are reported here. Nestlings in 2020 and 2021 were analyzed for all 40 PFAS.

Detection limits for other contaminants include total PBDEs <0.5 pg/g wet wt. and <0.15 for total PCBs ng/g wet wt. For the pesticides reported in depth in this paper, DLs were <0.06 ng/g wet wt. for p,p′DDE, p,p′DDD, cis- and trans-nonachlor, and hexachlorobenzene, 0.25 ng/g for dieldrin, 0.08 ng/g for mirex, and 0.15 ng/g for pentachlorobenzene. Refer to Supplementary Table S3 for the full list of organochlorine contaminants and their detection limits.

Trace elements were analyzed in liver tissue by the Trace Element Research Laboratory, College Station, TX, USA. In brief, samples were dried, digested and then analyzed by inductively coupled plasma optical emission spectroscopy for arsenic (As), cadmium (Cd), lead (Pb), and selenium (Se), and by inductively coupled plasma optical emission spectroscopy for silver (Ag), aluminum (Al), barium (Ba), beryllium (Be), boron (B), cobalt (Co), chromium (Cr), copper (Cu,) iron (Fe), magnesium (Mg), manganese (Mn), molybdenum (Mo), nickel (Ni), strontium (Sr), vanadium (V), and zinc (Zn). Mercury (Hg) was analyzed by direct analysis using atomic absorption from the fold-coated column. Detection limits were <0.5 µg/g dry wt. except for Al (0.9 µg/g) and Mg (1.73 µg/g, Supplementary Table S4). Average percent recovery was 94% and ranged between 82.6% and 110.6%, except that lithium had 0% recovery (Supplementary Table S4). Refer to Custer et al. [22] for complete methods.

For conversion of wet weight (wet wt.) concentrations to other bases (dry or lipid-normalized weights), use the following means: percent moisture for eggs, nestlings, and diet at 82.4%, 69.9%, 71.1%, respectively [23]. Mean percent lipid was 87.3%, 8.44%, and 2.38% for eggs, nestlings, and diet, respectively [23]. Moisture content of livers was 71.7% (n = 63, this study). Percent moisture was calculated as the difference between wet weight of the sample and the weight after it was freeze dried. Lipid content was measured gravimetrically. A subsample of the extract was weighed and allowed to evaporate. It was then baked at 105 °C for 30 min and the weight of the dried extract was recorded. The percentage of lipid in the extract was calculated.

### 2.2. Biomarker Assessments

Triiodothyronine (T3) and thyroxine (T4) were analyzed both in plasma and in the thyroid gland. The AccuBind T3 enzyme-linked immunosorbent assay (ELISA) kit and the Neonatal-T4 AccuBind ELISA kit (Monobind Inc., Lake Forest, CA, USA) were used for total T3 and T4 in plasma. Modified manufacturer’s protocols were followed so that the samples and standards were assayed in identical plasma-serum matrices. Thyroid glands were weighed in the laboratory (0.0001 g) and then digested with Pronase solution (Calbiochem, Millipore Sigma, Burlington, MA, USA), extracted with ethanol [24], and analyzed using the above kits for T3 and T4. Samples were analyzed in duplicate and means of the two were used in subsequent summary and statistical analyses. Concentrations are expressed as ng/mL (plasma) and ng/mg of tissue (glands).

For oxidative stress assessments, frozen livers were homogenized with stainless steel beads in a microcentrifuge tube at 4 °C in 1× phosphate-buffered saline (PBS, pH 7.4; FisherBioReagents, Waltham, MA, USA) at a concentration of 100 mg/mL. Following centrifugation for 10 min at 4 °C, the supernatant was diluted to 25 mg/mL in 1× PBS and frozen at −80 °C. Reduced glutathione (GSH) and oxidized glutathione (GSSG) were assayed at 8.33–12.5 mg/mL, thiobarbituric acid reactive substances (TBARS) were assayed at 12.5–25 mg/mL, and total sulfhydryl (TS) was assayed at 8.33–12.5 mg/mL. Protein bound sulfhydryl (PBSH) levels are the difference between TS and GSH. Data are presented as µmol/g of tissue.

Cell-to-cell variation in nuclear deoxyribose nucleic acid (DNA) content was assessed in red blood cells using flow cytometry with methods modified from Vindelov and Christensen [25] and Bigorgne et al. [10]. In brief, cells were lysed, stained with propidium iodide, and the DNA content in 10,000 cells was measured by florescence. Each sample was run twice, and the average coefficient of variation (CV) was calculated for each individual sample.

Two new biomarkers were developed to assess possible effects on immune function. Total immunoglobulin Y (IgY) concentrations were determined by enzyme-linked immunosorbent assay (ELISA) as described in Fassbinder-Orth et al. [26], with minor modifications. Standards were prepared using purified chicken IgY (Bio-Rad, Hercules, CA, USA; PAP001). The chicken IgY was serially diluted in coating buffer, pH 9.6 (Sigma Chemical, St. Louis, MO, USA; C3041) and standards ranged from 2.5 to 30 ng/mL. Tree swallow plasma was serially diluted 1:10,000 in coating buffer and vortexed briefly. Duplicate 75 µL aliquots of each sample were added to a flat-bottomed 96-well Nunc MaxiSorp™ plate (Thermo Fisher, Waltham, MA, USA), and incubated overnight at 4 °C. The coating solution was removed the following day after a 10-min incubation at room temperature and plates washed three times with Tris Buffered Saline, with Tween™ 20, pH 8.0 (washing buffer; Sigma, T9039). Blocking buffer (Tris Buffered Saline, with BSA, pH 8.0; Sigma, T6789) with 0.05% Tween™ 20 was then added to each well (200 µL/well). The covered plate was incubated at room temperature for 30 min with gentle shaking, followed by four washes. Each well then received 100 µL of polyclonal goat anti-bird IgY-HRP conjugated antibody (Bethyl Laboratories, Montgomery, TX, USA; A140-110P) diluted 1:10,000 in blocking buffer and the plate incubated at 37 °C for 1 h. After four additional washes, 100 µL of tetramethylbenzidine (TMB)-peroxidase substrate (Bethyl Laboratories) was then added to each well and the plate incubated in the dark at room temperature for 9.5 min. The reaction was stopped with 100 µL of 0.18 M H2SO4. Plates were read immediately on a BMG FLUOstar Omega Microplate Reader (BMG Labtech, Ortenburg, Germany). Absorbances were determined at 450 and 630 nm and the difference was used in a 4-parameter logistic curve to calculate the IgY concentration. Linearity and intra- and inter-assay variability, and analysis of blanks were determined for quality control. Two reference pools of multiple tree swallow plasma samples were used across plates. The mean intra-assay variability (%CV) was 3.8% and mean inter-assay variability was 13.6%. The assay reporting limit was 2.5 ng/mL, and the DL was 0.34 ng/mL.

Haptoglobin-like activity (PIT54), the second of the two immune biomarkers, was quantified colorimetrically using the Tridelta PHASE Haptoglobin Assay (TP801; Tri-Delta Development Ltd., Kildare, Ireland), following the manufacturer’s protocol at half volume. Briefly, plasma was thawed on ice and centrifuged for 10 min at 10,000× *g*. A 20 µL aliquot was then mixed with 5 µL of diluent (PBS, pH 7.6) and 8 µL of the mixture was pipetted in duplicate onto a flat-bottomed half volume 96-well plate (Costar). Standards, ranging from 0.039 to 0.625 mg/mL were also added in duplicate. Stabilized hemoglobin solution (Reagent 1; 50 μL) was added to each well and the plate was gently mixed and read at 580 nm on a BMG FLUOstar Omega Microplate Reader. Chromogen solution (Reagent 2; 70 μL) was then added to each well and the plate incubated at room temperature for 5 min before determining absorbance at 580 nm. Absorbance at 580 nm for Reagent 1 was subtracted from that for Reagent 2, and the difference analyzed by 4-parameter curve fit using MARS analysis software 2875A, V4.01 (BMG Labtech, Cary, NC, USA). Quality assurance/quality control was as described above for IgY. Two pooled tree swallow plasma samples were used to assess inter-assay variability. The mean intra-assay variability (% CV) was 5.4% and mean inter-assay variability was 10.0%. The assay reporting limit was 0.039 mg/mL, and the DL was 0.031 mg/mL.

### 2.3. Statistical Analyses

Chemical concentration data were first log-transformed to meet homogeneity of variance assumptions and then initially analyzed using 2-way analysis of variance (ANOVA, year, site and interaction) for individual PFAS at sites that had multiple years of information. Biomarker data were similarly analyzed but were not log transformed. Two geographic regions were established plus the reference lake, to understand larger geographical patterns based on hydrological connections and potential for transport of PFAS; these regions were also statistically compared. The North region included ANG, Martin Road, and Rice Lake North sites; it did not include the UMD site, while the St. Louis River region included the sites along that river. Because only 11% of 2-way ANOVAs (n = 18 tests total) for PFAS had a significant year effect, and because not all sites had multiple years of data, years (2019–2022) were combined for subsequent 1-way ANOVAs comparing among sites. Only those PFAS with >50% of the values greater than the DLs were statistically analyzed with ANOVA. For those PFAS that were statistically analyzed, for all values below the DL, one-half of the DL was substituted for subsequent ANOVA analyses and when calculating geometric means and 95% confidence intervals (CIs). Total_13_, Total_33_, and Total_40_ PFAS are the sum of either 13 (2019 data), 33 (2020), or 40 PFAS (2021), respectively, for which there were detectable concentrations. Total carboxylic acids (n = 7) and total sulfonates (n = 3), which were detected in >50% of egg or nestling samples, were also summed to generate a ratio of total carboxylic acids/total sulfonates. Pearson’s correlations were used for regression analyses to assess pairwise associations among chemicals and biomarker responses, as well as to correlate PFAS_13_ with PFAS_40_.

Accumulation rate of PFHxS and PFOS, the two most common PFAS, from eggs to 12-day old nestlings (11- to 13-day-old range) was calculated following Custer and Custer [27]. These two were the most common PFAS present in early AFFF formulations. In brief, the total masses (ng, concentration multiplied by sample wt.) in the egg and nestling were calculated for PFHxS or PFOS. Then, the mass in the egg was subtracted from the mass in the nestling, and that sum was divided by the age of the nestling. The slope of the line is the accumulation rate on a daily basis (ng/day). When no eggs were collected from the same clutch from which the nestling was collected, then the average mass of PFAS in eggs at that site was used. Sites were compared with 1-way ANOVA on non-transformed data.

Multivariate analyses were performed on standardized values (percentage of Total_40_ PFAS that each PFAS comprised). One-half of the DL was not substituted for samples below the detection limit, but a zero value was used for those analytes. The analyses included nonmetric multidimensional scaling plots (NMDS) for visualization of the data and analysis of similarity (ANOSIM) to test for differences among sites. Analysis of similarity calculates P- and R-values, which indicate whether differences are statistically significant and the degree of difference among groups. Differences in patterns are evident when R is >0.4, there is some support for pattern differences when R is between 0.3 and 0.4, and very little evidence for differences when R is <0.3 [28]. To reduce the number of zeros, only individual PFAS present in at least two samples across the entire data set were included in the multivariate analyses. The ANOSIMs were performed using Bray Curtis resemblances on standardized and then log-transformed data. To further examine patterns of PFAS exposure, the percentages that individual PFAS were composed of PFAS_40_ were calculated. To do this, the percentages that each PFAS contributed to total PFAS_40_ were calculated for each individual sample (eggs or nestlings) and then averages by site were calculated.

## 3. Results

### 3.1. PFAS Exposure

Ten of 40 PFAS were never detected in tree swallow tissues or diet (Table 1). These included N-EtFOSA, N-MeFOSE, HFPO-DA, ADONA, 9Cl-PF3ONS, 11Cl-PF3OUdS, 3:3 FTCA, PEESA, PFMBA, and NFDHA. Fourteen PFAS were detected in >50% of either egg, nestling, or diet samples. For the 27 PFAS not analyzed every year, the percentages in Table 1 were calculated, including only the years for which they were analyzed. Eggs had the largest number of different PFAS detected (n = 12) in >50% of samples, followed by nestlings (n = 9) and diet (n = 5). The 16 PFAS detected in between 1% and 50% of samples are listed in Supplementary Table S1, including the number detected. Four PFAS, PFOS, PFHxS, PFOA, and PFNA, were found with high frequency in all three matrices, while PFAS with 10 carbons or larger were detected less frequently in diet compared to swallow tissues (Table 1). Only 6:2 FTS, which is used in food wrappers and metal plating, was found in >50% of diet samples, but in <50% of other avian tissues.

**Figure 2:**
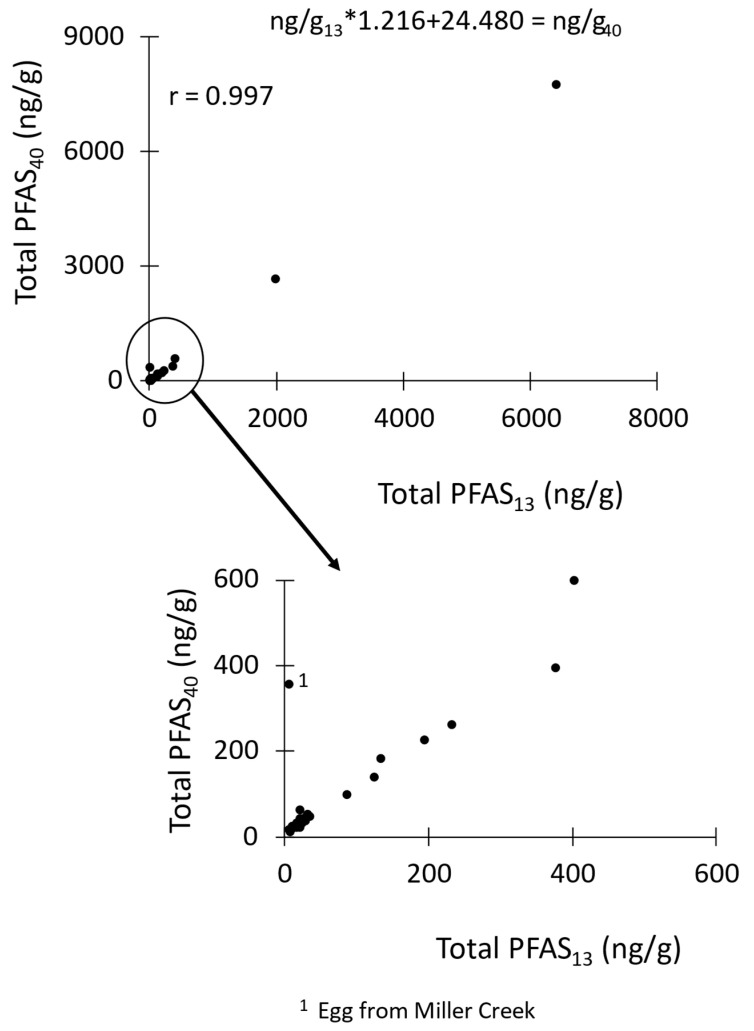
Correlation between original 13 (PFAS_13_) and current 40 (PFAS_40_) per- and polyfluoroalkyl substances (PFAS) in tree swallow eggs from the Duluth, MN area in 2021. Lower panel is an enlargement of the lower end of the distribution.

The correlation between total PFAS_40_ and the original total PFAS_13_, using data from 2021 when both PFAS subsets were analyzed in the same samples, was r = 0.997 (Figure 2) in eggs and r = 0.910 in nestlings (Supplementary Figure S1).

Perfluorooctane sulfonate was also highly correlated with PFAS_40_ (r = 0.997) and with PFAS_13_ (r = 0.999) because of the high proportion that PFOS contributes to biotic tissues. Based on the relationship from 2021, estimates for total PFAS_40_ concentration from any earlier work (i.e., with only 13 PFAS analyzed) can be made with the following formulas:

(1) PFAS_40_Estimate = 1.216 × PFAS_13_ + 24.48 for eggs
(2) PFAS_40_Estimate = 1.039 × PFAS_13_ + 7.005 for nestlings

For the reverse conversions, the following formulas can be used.

(3) PFAS_13_Estimate = 0.820 × PFAS_40 −_ 19.017 for eggs
(4) PFAS_13_Estimate = 0.876 × PFAS_40_ − 2.433 for nestlings

These slopes and intercepts describe the relationship within this data set and may, or may not, apply to data from other locations or for other species.

There were significant differences in PFAS concentrations in both eggs and nestlings among study sites (Table 2 and Table 3 for graphical presentation and Supplementary Figures S2 and S3), but also significant differences when sites were grouped by region. The sites in the North region near the airport, specifically the ANG and Rice Lake North, had the highest concentrations of PFOS and PFHxS in both matrices. The reference site, Boulder Lake ~15 km even farther to the north, always had significantly less PFAS than all other sites, with the exception of UMD. Egg PFAS concentrations at the North region sites were 30–40 times higher than at the reference lake, while nestling PFAS concentrations were 10–15 times higher than at the reference lake. This contrasts with sites along the St. Louis River, which is an urban river with mainly non-point PFAS sources. Along the St. Louis River sites, PFOS and PFHxS were perhaps slightly elevated, at 2–5 times higher than the reference lake, albeit not significantly so in eggs and nestlings.

**Figure 3:**
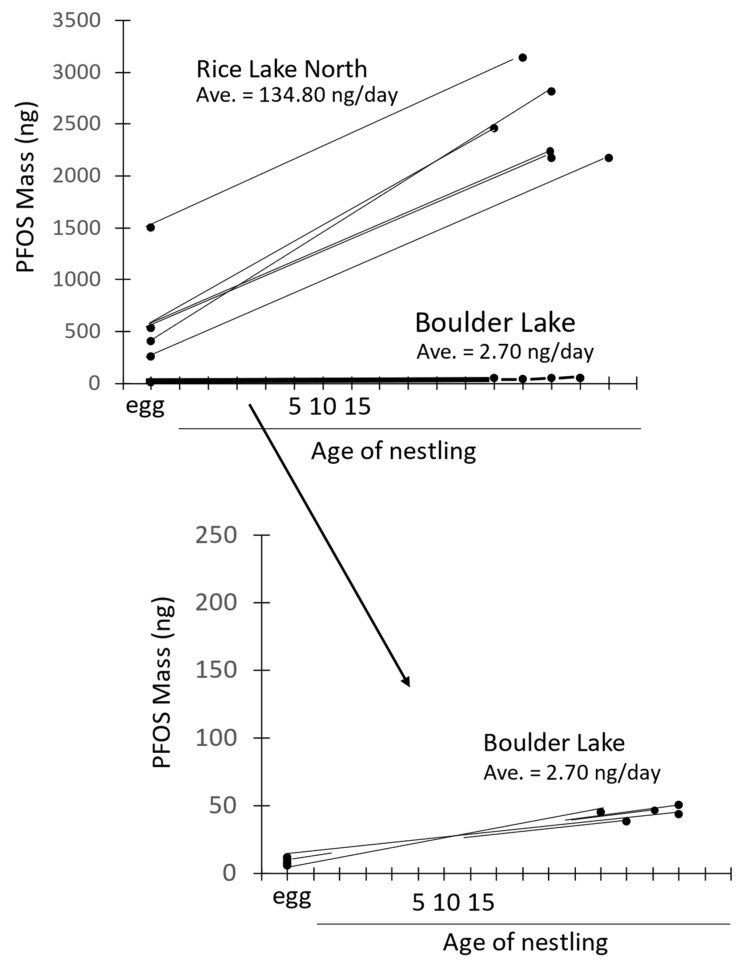
Total mass (ng) of perfluorooctane sulfonate (PFOS) in pairs of eggs and sibling 12- to 15-day old nestlings at Rice Lake North and the reference site (Boulder Lake, upper panel) in the Duluth, MN area in 2020–2021. Lower panel is expanded to provide more detail at the reference lake. Average accumulation rates (ng/day) are provided for the two sites.

Perfluorohexane sulfonate, the second most common PFAS in historical AFFF formulations [29], had 180–260 times higher concentrations at the North region sites compared to the reference lake in eggs and 32–49 times higher concentrations in nestlings. The difference between the reference lake and St. Louis River sites for PFHxS was only 2–3 times, except for Boy Scout Landing, where PFHxS was 8 times higher in nestlings. Differences were few among any of the sites for the carboxylic acids in either the eggs or nestlings (Table 2 and Table 3). Average accumulation rate (ng/day) of PFOS was ~10 times higher at the North region sites than along the St. Louis River (Table 4) and ~50 times higher than at the reference lake. The slopes of the lines (=accumulation rate) are graphically shown by plotting the total mass of PFOS (ng) in the egg/nestling pairs from Rice Lake North and the reference lake to show these differential accumulation rates (Figure 3). 

Accumulation rate for PFHxS was ~13 times higher at the North region sites compared to the St. Louis River and ~25 times higher than at the reference lake. The rate for PFHxS accumulated at Boy Scout Landing was 2–3 times higher than the other St. Louis River sites but was only significantly higher than at Thomson Reservoir and Miller Creek (Table 4). The site with the lowest accumulation rate was UMD with a rate of 1.19 ng/day for PFOS and 0.16 ng/day for PFHxS.

**Figure 4:**
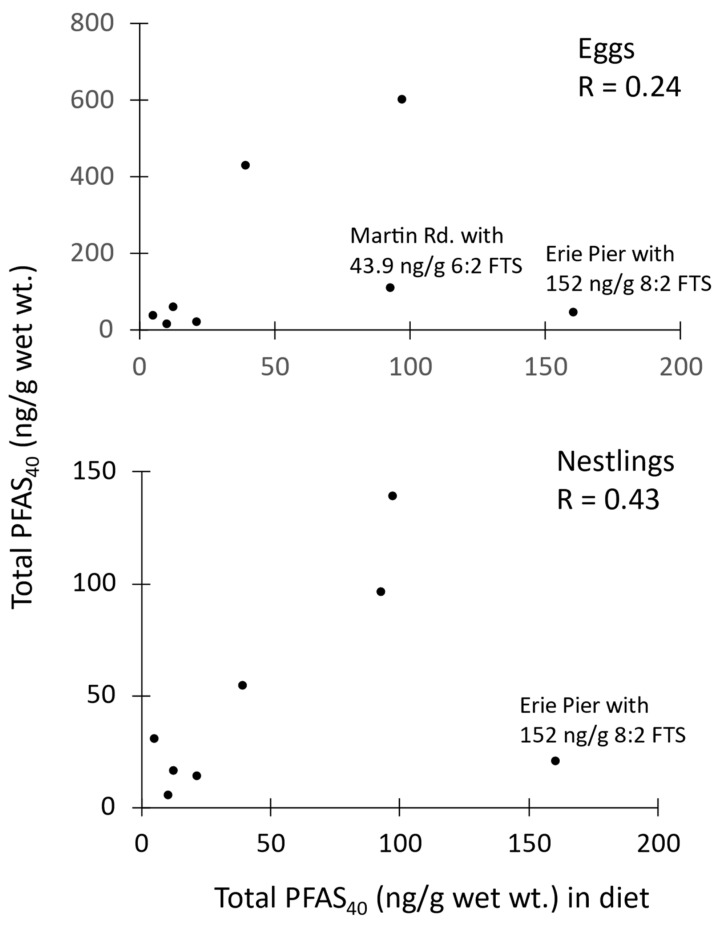
Correlation between the sum of 40 per- and polyfluoroalkyl substances (PFAS_40_) in diet and nestling carcasses (**upper**) and diet and eggs (**lower**) from the Duluth, MN area in 2020–2021.

Concentrations in diet samples (Table 5), generally one composited sample per site, were dominated by PFOS at all sites except at Erie Ponds, where 8:2 FTS was found at the highest concentrations. Different also from other sites was Martin Road, where 6:2 FTS concentrations were virtually identical to PFOS concentrations. Total PFAS_40_ in diet tracked the concentrations in eggs and nestlings (Pearson’s r = 0.4 and r = 0.24, Figure 4). The one or two data points that did not conform with this relationship were at sites with unusual concentrations of 8:2 FTS and 6:2 FTS present in diet samples. These two PFAS are rarely detected in swallow eggs (0–18%) or nestlings (0–5%, Supplementary Table S1), which accounts for them appearing as ‘outliers’ in the regression. PFAS_13_ was an even better fit between diet and either nestlings (r = 0.95) or eggs (r = 0.85) because the 13 analytes did not include either 8:2 nor 6:2 FTS.

### 3.2. PFAS Composition—Regional and Site Comparisons

**Figure 5:**
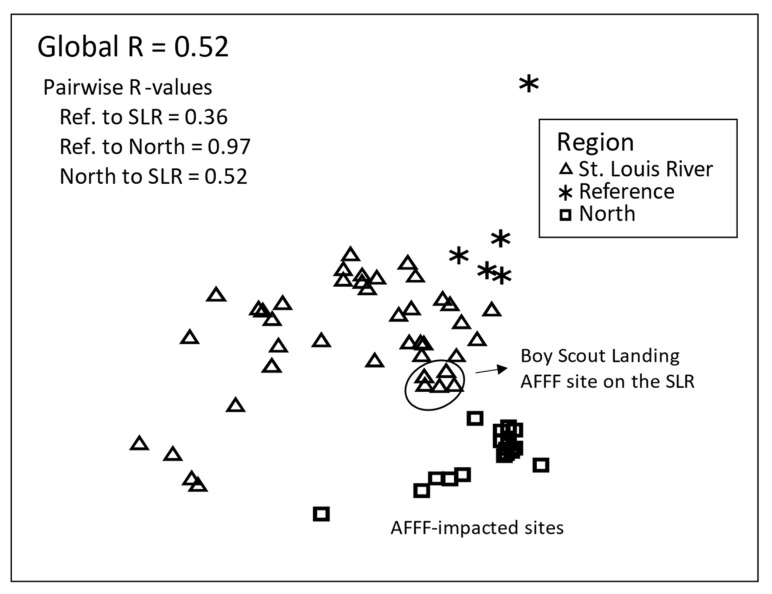
Nonmetric multidimensional scaling (NMDS) plot of per- and polyfluoroalkyl substances (PFAS) in tree swallow nestlings (2010 and 2021) by region in the Duluth, MN area for n = 25 PFAS detected in ≥2 samples. Note: NMDS plots are unitless, AFFF = aqueous film forming foam, SLR = St. Louis River.

Twenty-one and 25 PFAS were detected in at least two egg or nestling samples and were included in the multivariate assessments that focused on regional and individual site comparisons. There were significant differences among regions (Global R = 0.52) in the types of PFAS present in tree swallow nestling tissues (Figure 5) and to a lesser extent in tree swallow eggs (Global R = 0.45, Supplementary Figure S4). Note that NMDS plots do not have units. In nestlings, there was almost complete separation (R = 0.97) between the North region sites and the reference site and a substantial difference between the North region sites and the St. Louis River sites (R = 0.52). The reference site and the St. Louis River sites differed little in PFAS composition (types of PFAS present, R = 0.36) for nestlings. Boy Scout Landing, a site along the St. Louis River, but near a fire training facility, was intermediate in multidimensional space between the St. Louis River and the AFFF-impacted North region sites (circled in Figure 5). In eggs, the separation was similar to that for the nestlings, i.e., the North region was separated from both the reference (R = 0.94) and St. Louis River (R = 0.51) regions, and the reference and St. Louis River regions were similar (R = 0.11, Supplementary Figure S4). The egg samples from Boy Scout Landing were intermediate in multivariate space between the North region and the St. Louis River region.

On an individual site basis, the composition of PFAS in nestling carcasses differed among all sites in multivariate space (ANOSIM, R = 0.814, Supplementary Figure S5) except for between Martin Road and Rice Lake (R = 0.40), and between Martin Road and the ANG (R = 0.34) sites. Geographically, Martin Road is midway between Rice Lake and ANG, which probably explains those similarities. For eggs, there was less separation among sites (R = 0.573, Supplementary Figure S6). Thomson, the farthest upstream site on the St. Louis River and located in more pristine habitat, did not separate from the reference lake (R = 0.06). Other observations were that Boy Scout Landing did not differ from Miller Creek (R = 0.07) or UMD, (R = −0.26), the ANG site did not differ from Martin Road (R = 0.02) or UMD (R = 0.22), and finally Miller Creek did not differ from UMD (R = 0.14) or Erie Ponds (R = 0.18, Supplementary Figure S6). Often the sites that did not differ were adjacent to one another geographically. The more terrestrial site at UMD also differed from more aquatic-oriented sites in PFAS profiles. The separation in multivariate space for most individual sites, both nestlings and eggs, indicates that very subtle differences among sites can be described with multivariate analyses, which can also include an element of stochasticity in the characterization.

**Figure 6:**
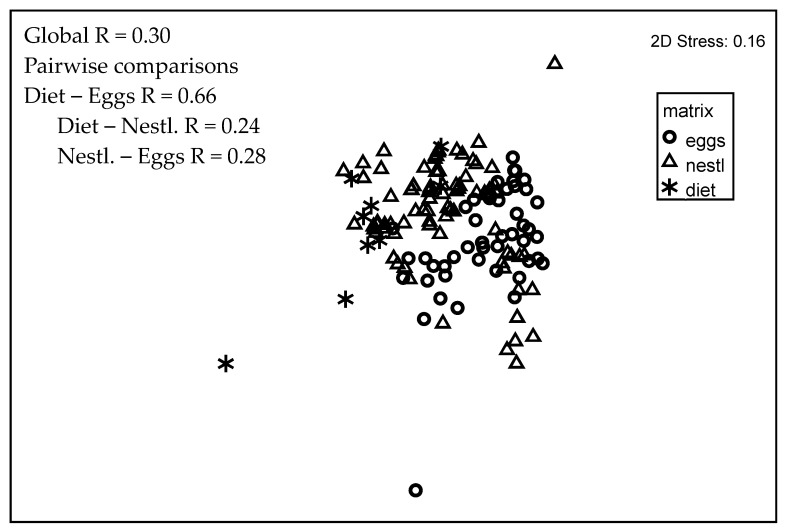
Nonmetric multidimensional scaling (NMDS) plot of per- and polyfluoroalkyl substances (PFAS) in tree swallow egg, nestling, and diet samples (2010 and 2021) in the Duluth, MN area for n = 27 PFAS detected in ≥2 samples in at least one of the three matrices. Note that NMDS plots are unitless.

Comparing the composition of the 27 PFAS detected in at least two egg, nestling, or diet samples, there was little difference in multivariate space among the three matrices (Global R = 0.30, Figure 6). Diet was most dissimilar to the eggs (pairwise R = 0.66), but the PFAS profile in diet and nestlings (R = 0.24) and eggs and nestlings (R = 0.28) could not be distinguished.

### 3.3. Contribution of Individual PFAS to Total PFAS

**Figure 7:**
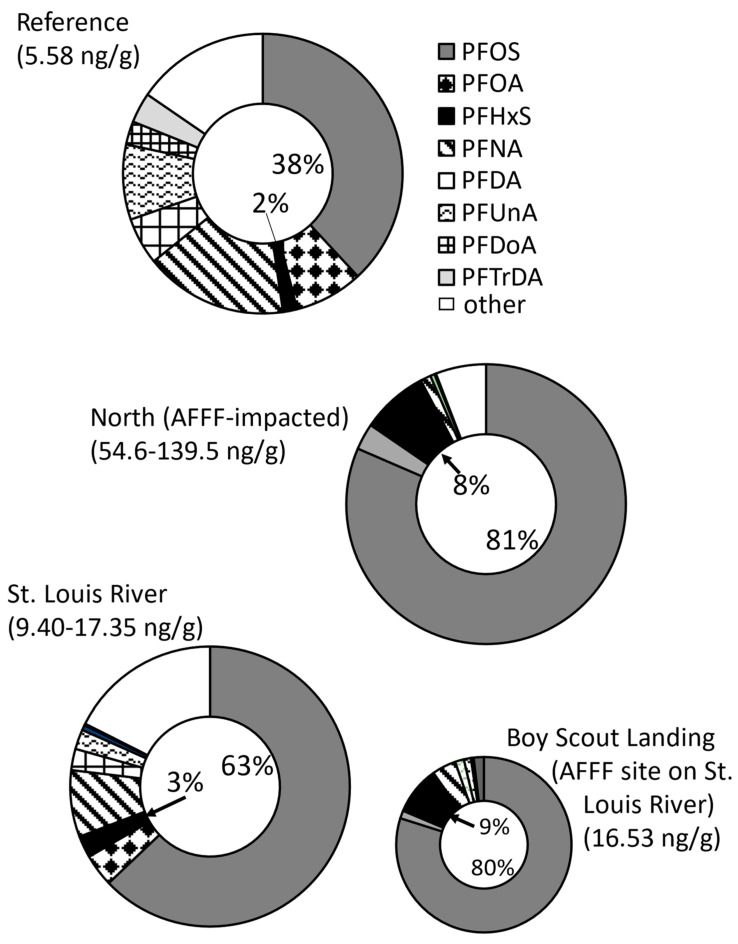
Average percentage of 40 per- and polyfluoroalkyl substances (PFAS_40_) that individual PFAS comprised in tree swallow nestling carcasses in the three regions near Duluth, MN 2020–2021. St. Louis River includes Boy Scout Landing which has an aqueous film forming foam (AFFF) source and has been pulled out for visualization. Percentages inside circles are for perfluorooctane sulfonate (PFOS) and perfluorohexane sulfonate (PFHxS). Concentrations or range of total PFAS_40_ concentrations are provided for sites within each region in parentheses. Note: AFFF = aqueous film forming foam, PFOA = perfluorooctanoate, PFNA = perfluorononanoate, PFDA = perfluorodecanoate, PFUnA = perfluoroundecanoate, PFDoA = perfluorododecanoate, PFTrDA = perfluorotridecanoate.

Examining the percentage of total PFAS_40_ that individual PFAS comprised in tree swallow nestling carcasses at the AFFF-impacted North region sites, PFOS averaged 81% of total PFAS_40_ followed by PFHxS at 8% (Figure 7). These percentages contrasted with 38% (PFOS) and 2% (PFHxS) at the reference site, and 63% (PFOS) and 3% (PFHxS) at St. Louis River sites. Boy Scout Landing, a site along the St. Louis River, had PFAS composition, which was nearly identical to the North AFFF-impacted sites (80% and 9% for PFOS and PFHxS). The reference site had qualitatively larger percentages of six other PFAS, including PFOA, PFNA, PFDA, PFUnA, PFDoA and PFTrDA, compared to the North region sites, but differed to a lesser degree when compared to the St. Louis River sites, which also had a larger number of other PFAS compared to the North region.

**Figure 8:**
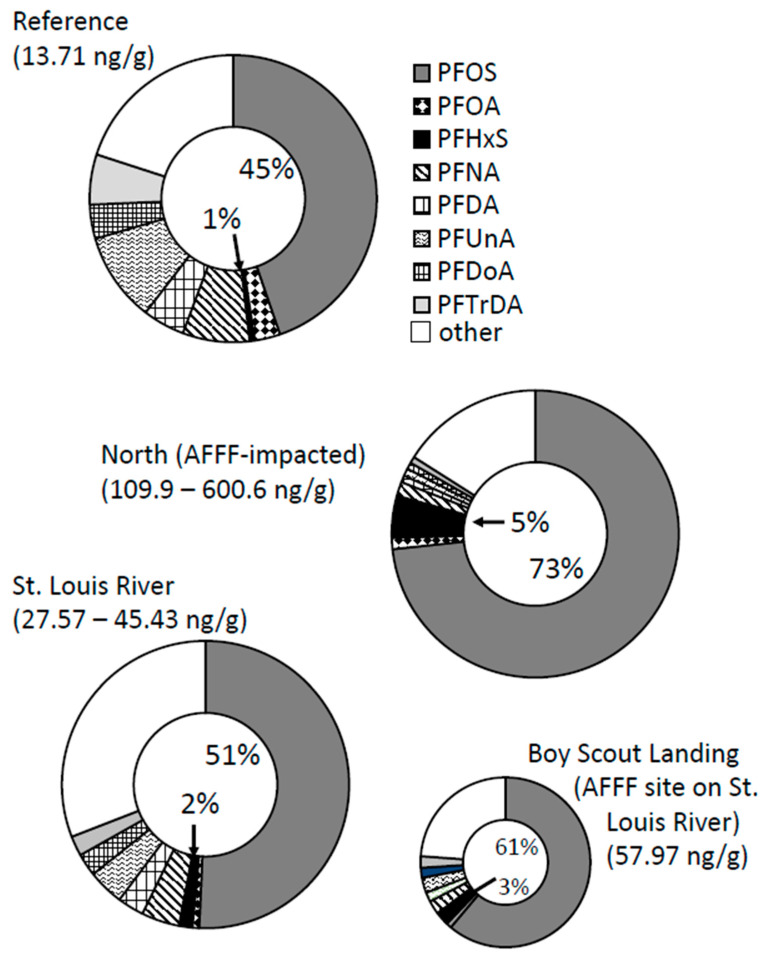
Average percentage of 40 per- and polyfluoroalkyl substances (PFAS_40_) that individual PFAS comprised in tree swallow eggs in the three regions near Duluth, MN 2020–2021. St. Louis River includes Boy Scout Landing, which has been pulled out (lower right) for visualization. Percentages inside circles are for perfluorooctane sulfonate (PFOS) and perfluorohexane sulfonate (PFHxS). Concentrations or range of total PFAS_40_ concentrations are provided for sites within each region in parentheses. Note: AFFF = aqueous film forming foam, PFOA = perfluorooctanoate, PFNA = perfluorononanoate, PFDA = perfluorodecanoate, PFUnA = perfluoroundecanoate, PFDoA = perfluorododecanoate, PFTrDA = perfluorotridecanoate.

The proportion of different PFAS in tree swallow eggs (Figure 8) had similar profile differences among regions as that found in nestlings (i.e., a higher percentage of PFOS and PFHxS) at AFFF sites and a higher percentage of many other PFAS at the reference site. Similar also to the proportions in nestlings, the St. Louis River was more similar to the reference percentages except for Boy Scout Landing with its AFFF source. The Boy Scout Landing site more resembled the North region AFFF-impacted sites in spite of total PFAS_40_ concentrations being less than half than that found at the North region sites.

### 3.4. Carboxylic Acid to Sulfonate Ratios

**Figure 9:**
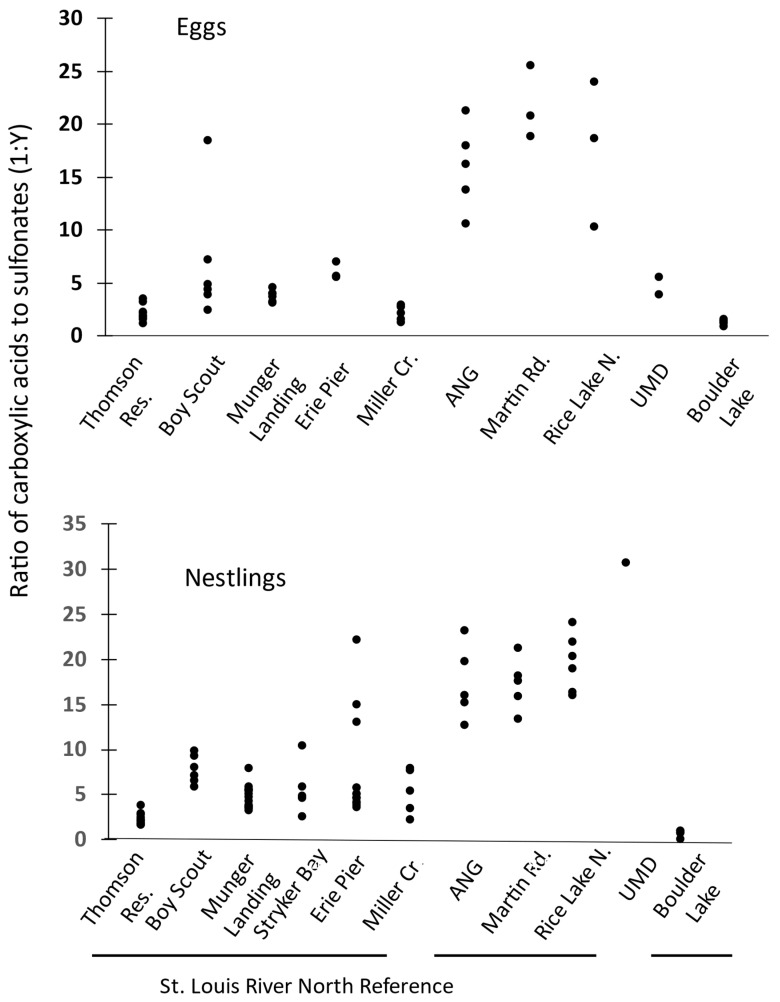
Ratio of total carboxylates to total sulfonates in tree swallow eggs (**upper**) and in nestlings (**lower**) at sites in the three regions near Duluth, MN, 2020–2021.

The sum of seven carboxylic acids and three sulfonates found in >50% of eggs and nestlings (Table 2 and Table 3) were converted to ratios and plotted by site (Figure 9). In the North region, with a putative AFFF source, the ratio was always >1:10 in both eggs and nestlings (Figure 9) whereas, along the St. Louis River, the ratios were generally below the 1:10 threshold. Boy Scout Landing site did show some evidence of the AFFF source at the Lake Superior College Emergency Response Training Center with one of six ratios in eggs, 1:18, indicating an AFFF source. There was no indication of an AFFF source in the nestlings. The reference location had ratios consistently less than 1:1 in nestlings and less than 1:1.5 in eggs.

### 3.5. Organochlorine and Trace Element Contaminants

For total PCBs and the organochlorine insecticides, there were few differences among the sites (Supplementary Table S5). Total PCBs in nestling carcasses were significantly higher at Boy Scout Landing (34.15 ng/g wet wt.) than at the reference lake (7.50 ng/g), but neither site was different than all other sites with geometric means that ranged between 8.77 ng/g (Rice Lake North) and 27.92 ng/g (ANG). There were no statistical differences among sites for total PBDEs (lowest = 1314 pg/g [UMD] and highest = 3008 pg/g [Boy Scout Landing]), or for p,p′DDE (4.19 ng/g [Boy Scout Landing] and 6.89 ng/g [UMD]), dieldrin (0.09 ng/g [ANG and Rice Lake North]) and 0.67 ng/g [UMD]), or mirex (0.20 ng/g [Rice Lake North] and 0.41 ng/g [UMD]). The reference lake had significantly lower concentrations than at UMD for pentachlorobenzene (0.12 vs. 0.41 ng/g) and for trans-nonachlor (0.18 vs. 1.79 ng/g). Hexachlorobenzene, however, was significantly lower at UMD (0.26 ng/g) than at the other sites, except for ANG (0.33 ng/g) and Martin Road (0.33 ng/g, Supplementary Table S5). Organochlorine contaminants in two egg samples were all at background concentrations (Supplementary Table S6)

Ten of 22 trace elements were detected in >50% of tree swallow liver samples (Supplementary Table S7) and included Cd, Co, Cu, Fe, Hg, Mg, Mn, Mo, Se, and Zn. The other trace elements were detected in <33% of samples and included Al, As, Ba, Be, B, Cr, Pb, Li, Ni, Sr, Tl, and V (Supplementary Table S4). There were few differences in trace elements among sites (Supplementary Table S7). Cadmium was significantly higher at the ANG (0.07 μg/g dry wt.) compared to Boulder Lake (0.03 μg/g), Martin Road (0.04 μg/g), and Munger Landing (0.04 μg/g). Mercury had significantly higher concentrations at Boy Scout Landing (0.18 μg/g dry wt.) than at Rice Lake North and Martin Road (both 0.10 μg/g) and ANG and Miller Creek (both 0.07 μg/g). Selenium, a third trace element that can have negative effects in wildlife [30], was significantly higher at Thomson Reservoir (4.54 μg/g dry wt.) than all other sites except for the two sites immediately downstream of that site (Munger Landing [3.43 μg/g] and Stryker Bay [3.61 μg/g] Supplementary Table S7). The essential trace elements, such as copper and zinc, showed few statistical differences among sites.

### 3.6. Biomarkers

While six of eight oxidative stress biomarkers statistically differed (*p* < 0.05) among sites, there were few actual differences among sites (Table 6). For example, PBSH at UMD and Miller Creek sites were significantly lower than at Erie Ponds in 2019, but those two sites were not different from Erie Ponds in 2021. Miller Creek and UMD also had lower PBSH than Stryker Bay in 2019. The other 11 sites did not differ from one another. Another example is for thiol, for which Erie Ponds in 2019 was significantly higher than Miller Creek or UMD, with all the other sites being statistically similar to one another. Neither of the two immune responses, IgY and PIT54, nor T3 or T4 in plasma differed among sites (Table 7). Both thyroid hormones in glands differed slightly among sites, although for T3 the means did not separate with Bonferroni’s mean separation tests. Thyroxine (T4) in the thyroid gland was significantly lower at ANG and Miller Creek compared to Rice Lake North and at Thomson Reservoir in both years. Ethoxyresorufin-O-dealkylase activity was significantly elevated at Munger Landing in 2019 compared to most other sites, including that same site in 2021. The sites that Munger Landing in 2019 did not statistically differ from were Thomson Reservoir in 2019, Stryker Bay, and Erie Ponds in both years. (Table 7). There was no indication of differential distribution of DNA in the nuclei of red blood cells among sites, i.e., DNA CV did not differ among sites (Table 7).

## 4. Discussion

### 4.1. PFAS Exposure

Time has not diminished the degree of PFAS exposure in tree swallows in this part of Minnesota. Because plasma and egg concentrations are similar [21,31], comparisons can be made with some confidence with earlier St. Louis River data that were assessed in plasma [8]. Concentrations of PFOS at Rice Lake North (469.7 ng/g in eggs) have not materially changed since the area was sampled in 2010–2011 (498 ng/g in nestling plasma, [8]. Note that Rice Lake North and Wild Rice (site name from an earlier study) receives water from the vicinity of the Duluth International Airport/ANG via subsurface water and small creeks entering from the south. Similarly, the middle stretch of the St. Louis River (Stryker Bay, 12.2 ng/g in 2010–2011, [8]) also had similar concentrations of PFOS as at Munger Landing area (16.31 ng/g) in the current study. The lower reaches of the St. Louis River, however, seemed to show a considerable reduction in PFOS since 2010–2011 (114–118 ng/g) compared to the current study at Miller Creek and Munger Landing (6.52 and 16.31 ng/g). The reasons for this are unknown but may have to do with differences in the sites that were sampled, which differed between the two studies, or possibly the removal of sediment over the intervening years associated with navigational dredging and Great Lakes Restoration Initiative projects (St. Louis River Area of Concern USEPA [https://www.epa.gov/great-lakes-aocs/st-louis-river-aoc#restoration], 6 November 2024)

To our knowledge there have been no mitigation activities in the Duluth area for PFAS per se, which probably accounts for the lack of change in PFAS exposure in tree swallows between 2010 and the current study. This contrasts with the >50% reduction in PFOS and PFDS exposure observed in great blue heron (*Ardea herodias*) eggs between 1993 and 2010–2011 in the Minneapolis/St. Paul metro area [32]. Total PFAS also declined in bald eagle (*Haliaeetus leucocephalus*) nestling plasma along the Mississippi River in the Minneapolis/St. Paul metro area between 2006 and 2015 [33,34]. The reduction in both those studies is likely to have resulted from cessation of production of perfluoroalkyl sulfonates at a manufacturing plant in that area beginning in 2000.

Distinguishing different sources of PFAS in environmental media is a recurring need; methods are being developed and refined [35,36], especially for water. Source tracking of PFAS in abiotic environmental media is challenging because formulations may have changed through time, and the suite of PFAS present may be affected by environmental transformation and transport processes. Source tracking using biotic media is even more complicated because, in addition to the above, exposure pathways may differ among species, toxicokinetic differences can exist among biota, and a plethora of other factors can be operating [37]. One goal for assessing both biotic and abiotic media is to determine if there are sentinel (PFAS) compounds or other methods, such as ratios, that might be useful to create fingerprints for specific sources [35]. There are two putative direct sources for PFAS, specifically AFFF sources, in the greater Duluth area of Minnesota: (1) the joint civil–military airport located 9 km northwest of Duluth that is used by both the MN Air National Guard’s 148th fighter Wing, and the Duluth International Airport commercial traffic, and (2) Lake Superior College’s Emergency Response Training Center (LSC-ERTC). One indication of an AFFF source is higher absolute concentrations of certain PFAS in avian tissues because the input volume and frequency of use is often high, especially at training locations. At the ANG base and two areas to the north (Martin Road and Rice Lake North), PFAS_40_ concentrations were 5 to >10 times higher than the other sites sampled in this study (Table 2 and Table 3). The Boy Scout Landing site, which is downstream of LSC-ERTC, however, did not show elevated PFAS_40_ contamination in either eggs or nestlings compared to other sampling locations along the St. Louis River, as might be expected (Table 2 and Table 3). This might have occurred because the large volume of water in the St. Louis River would dilute much of the PFAS contamination potentially coming from the fire-training facility, or perhaps because the swallows were feeding on insects emerging from the less contaminated St. Louis River in addition to insects from the small, contaminated drainages in that area. Another reason that elevated total PFAS exposures are not always a reliable indication of an AFFF source is because PFAS could be coming from other sources. In addition to AFFF contamination, unusual elevation of total PFAS can be due to contamination associated with manufacturing plants (e.g., [31,38]). This may not be a widespread issue, however, because there are few manufacturing sites in the U.S or elsewhere. This highlights the limitations in using absolute concentration, rather than proportions of different PFAS (more in discussion below), which might offer another method, either alone or in combination, to identify an AFFF source.

A possible marker PFAS to distinguish AFFF usage from other sources is the concentration and proportion of PFHxS. This PFAS was the second most prevalent PFAS, after PFOS, in many of the earlier AFFF formulations [29]. Using this as a marker PFAS, the Boy Scout Landing site also showed the influence of an AFFF source, with PFHxS concentrations 2–6 times higher than the rest of the St. Louis River sites (Table 2 and Table 3). Additionally, the accumulation rate for PFHxS at Boy Scout Landing was three times higher than along the rest of the St. Louis River (Table 4). The three North region sites also showed a strong AFFF signature, with PFHxS concentrations 5–45 times higher than the St. Louis River sites, and between 63 and 188 times higher in eggs and 40–48 times higher in nestlings compared to the reference lake (Table 2 and Table 3). The average accumulation rate for PFHxS at the three North sites (9 ng/day) was 26 times higher than at the reference lake and 13 times higher than along the St. Louis River (Table 4). PFHxS was not detected in 1 of 4 nestling samples or in 2 of 5 egg samples at the reference lake and were always at low concentrations in those samples, just above the DL. Although some have attempted to use elevated PFOA as an AFFF marker, we find it is less useful for that purpose because PFOA is present at much lower concentrations in fire-fighting foams [29], and also because its wide use in many other commercial products makes it less useful as a marker for AFFF source identification.

The relative proportion of PFHxS to total PFAS, the proportion of other types of PFAS, or the ratio of PFHxS to PFOS can also be used to distinguish sources [35,36,37,38,39]. Proportions may show a clearer signature because they factor out absolute PFAS concentrations, which vary among locations and blur the assessment, especially at sites with low levels of PFAS contamination. In nestling carcasses, the proportion of PFOS and PFHxS were 81% and 8% at the three north AFFF-impacted sites, 80% and 9% at Boy Scout Landing, also an AFFF-contaminated site, but were only 38% and 2% at the reference lake and 63% and 3% at the St. Louis River sites (Figure 7). The percentages at the three North region sites were very similar to the proportions of PFOS and PFHxS in eggs at Clarks Marsh near Oscoda, MI, also an AFFF-contaminated site, where the proportions of PFOS and PFHxS were 87% and 7.3% [21]. A hallmark of other sources, including atmospheric deposition, is the presence of many other detectable PFAS. For example, six other PFAS comprised ≥2% of PFAS_40_ at the reference lake and four PFAS comprised ≥2% along the St. Louis River. This compared to only one other PFAS comprising >2% at the North AFFF-impacted sites (PFOA) or Boy Scout Landing (PFNA). At Oscoda, no other PFAS comprised >1% of total PFAS_13_ [21]. Expressed another way, the ratio of PFHxS:PFOS ranged between 1:25.1 and 1:46.6 in eggs and between 1:23.4 and 1:31.6 (nestlings) at non-AFFF sites along the St. Louis River compared to lower ratios of between 1:12.4 and 1:20.6 (eggs) and between 1:6.7 and 1:14.8 (nestlings, derived from data in Table 2 and Table 3). A ratio of <1:20 may indicate an AFFF source and a ratio >1:20 could indicate a non-AFFF source. This ratio was accurate for eggs (1:50.7) at the reference lake with background exposure to PFAS but did not accurately reflect a non-AFFF source at the reference lake for nestlings where the ratio of PFHxS/PFOS was only 1:6.2 (nestlings).

Another ratio, that of total carboxylic acids to total sulfonates, similar to methods used by Kibbey et al. [36] in water, also seemed to distinguish AFFF sources from other PFAS sources in our study (Figure 9). There was a distinct threshold at ~1:10 in eggs and nestlings. The reference location tended to have an even lower ratio of <1:1 (nestlings) or 1:1.5 (eggs). Kibbey et al. [36] found that the ratio of carboxylic acids to sulfonates in water correctly classified 91.7% of AFFF sources (accuracy). While the accuracy was less than that produced by three of four machine learning techniques they evaluated, the benefits of using simple proportions or ratios may be preferred by site managers, State and Federal regulators, and others without access to more statistically intensive methods.

A combination of these methods, concentrations of PFOS and PFHxS, ratio of PFHxS:PFOS, proportion of PFOS and PFHxS to total PFAS, ratio of carboxylic acids to sulfonates, and presence of many other PFAS, is useful for distinguishing AFFF sources in biota from the multitude of other PFAS source. Using PFHxS as a bona fide AFFF marker may soon become dismissed/obsolete because of the current, ongoing, forensic work that is identifying AFFF sources with high certainty based on fluorinated compounds not targeted by current PFAS analysis methods, such as those used in the present study (method 1633, [14,40]), as well as the development and more wide-spread usage of fire-fighting foams that do not contain PFAS. Additionally, concentration of PFHxS would not be useful in distinguishing the fluorotelomer-based AFFF formulations.

Accumulation rate of contaminants is a very powerful tool [27] to help understand local exposure because it factors out what is present in the eggs, and only reflects what is received from the local environment, primarily from diet. For tree swallows, the local feeding radius is < approx. 1 km [41]. Nestlings from Rice Lake North and Martin Road had in excess of 130 ng/day of PFOS accumulated per day (Figure 3, Table 4), which far exceeded that at sites along the St. Louis River (8.99–17.16 ng/day) or the reference lake (2.65 ng/day). Even at this relatively pristine area, some PFAS accumulation was occurring and was most likely derived from atmospheric deposition. Of note, the site with the lowest accumulation was UMD (1.19 ng/day), located in a primarily terrestrial setting, which may account for this low rate. Note that sample size was only a single egg-nestling pair at UMD, so those results should be interpreted with caution. Accumulation rate has been effectively used with other contaminants, such as PCBs [42,43]. This method is effective in quantifying differences among sites (this study) or changes through time as a result of remediation actions (e.g., [43]). This method only works, however, for contaminants that bioaccumulate in biotic tissues.

As in humans [5], diet is the dominant route of exposure for many chemical contaminants in tree swallows. Supporting this is the fact that tree swallows directly drink little water, so this is unlikely to be a major route of exposure. Therefore, much of the body burden of PFAS in nestling tree swallows comes from the insects they are fed, which are typically aquatic in origin [12,44]. This is consistent with modeling studies that characterized nestling tree swallow dietary uptake of PCBs from aquatic insects emerging from local contaminated sediment [45]. It has also been suggested that aquatic emergent insects propagate contaminant transfer across ecosystem boundaries to riparian and local terrestrial insect food webs [46], potentially exposing other insectivorous bird species that usually forage on terrestrial insects. In the current study, there is little separation in multivariate space between the types of PFAS present in diet versus those in nestling carcasses (Figure 6). There is more separation in multivariate space between diet and eggs (Figure 6), as might be expected by the physiological and chemical partitioning processes occurring in the female as food is assimilated to support oogenesis and yolk deposition. Finally, correlation was high (r > 0.85) on a site basis between concentrations of PFAS_13_ in the diet and those in both the eggs and nestlings (relationships not shown). For PFAS_40_, data were less well correlated because there were ‘outliers’ resulting from higher concentrations of some PFAS (6:2 FTS and 8:2 FTS primarily) that were found in diet samples, i.e., aerial stages of benthic aquatic insects, but which were rarely found in either eggs or nestlings (Figure 4).

Improvements in analytical chemistry resulted in having a progressively larger suite of PFAS analytes over time in the test samples. Nonetheless, we were able to compare across years due to a strong correlation (r > 0.95) for both eggs and nestlings between the PFAS_13_ and PFAS_40_ datasets (Figure 2). Additionally, about one-half (n = 7 of 13) of the individual PFAS detected in >50% of samples had been included in the PFAS analyte list for many years (since at least 2008 for tree swallow data), and those seven comprised >70% of the concentrations except at two sites, both at the lower end of the St. Louis River (Table 2 and Table 3). Erie Ponds had higher concentrations of 7:3 FTCA and PFDA than other sites, and Miller Creek had relatively lower concentrations of PFOS relative to other sites, which accounted for the lower percentage of PFAS_13_ vs. PFAS_40_ at these two sites (Table 2 and Table 3). It is straightforward to convert from PFAS_13_ to PFAS_40_ and vice versa based on the data in the current study (Figure 2). Note, however, that the slopes and intercepts generated by the current data set to make these conversions may not apply to other locations. Data from other situations would be needed to develop more robust conversion factors and evaluate the consistency of these relationships.

### 4.2. Other Chemical Exposures

In contrast to PFAS exposure, halogenated contaminants, such as total PCBs and PBDEs, seemed to be continuing to decline, albeit slowly, in tree swallow tissues since their use was banned in the late 1970s (PCBs) and when the phase-out of PBDEs began in the early 2000s. Total PCBs in nestling carcasses ranged between 12.2 and 112 ng/g in 2010–2011 along the St. Louis River and Wild Rice Lake (=Rice Lake North in the current study, [8]) and are between 7.5 and 34.25 ng/g at those two sites in the current study, so there is a modest diminution in concentration (Supplementary Table S5). Large changes in exposure might not be expected, however, because concentrations at all sites in the current study were already low, i.e., in the lowest 10th percentile of 66 Great Lakes sites [8], and would be considered background levels for PCBs. Total PBDEs have declined in nestling carcasses by more than one-half in the environments along the St. Louis River (3.0 ng/g) and in the larger Duluth area (1.3–2.0 ng/g, the current study, Table 6) compared to 8.0–16.8 ng/g in 2010–2011 [8]. Again, the current levels are in the lowest 5th percentile compared to the 68 sampled Great Lakes sites [8] and should also be considered background levels. Overall, concentrations of PBDEs and PCBs are below any known effect thresholds for hatching success and biomarker responses in tree swallows [11,23,47].

Likewise, trace element exposures were at what might be considered background levels (Supplementary Table S7). Mercury concentrations were between 0.7 and 0.18 μg/g dry wt. in the current study, nearly identical to the values found at other sites along the lower St. Louis River between 2010 and 2015 [22]. In that study, the St. Louis River sites were in the lowest 50th percentile of the 76 sites sampled across all five Great Lakes. Cadmium in the current study was similar to, or lower than, the four sites sampled earlier (2010–2015) in this drainage which ranged from 0.04 to 0.11 μg/g [22]. Concentrations in that analysis were 11th highest out of 39 Areas of Concern sampled, so were slightly elevated above background, but were nowhere near effects levels for adverse effects and lethality (40 μg/g, liver tissue) established by Furness [48]. Finally, Se concentrations in the current study were similar to those quantified earlier (2010–2015) along the St. Louis River, which were in the lower 25% quartile of all sites [22].

### 4.3. Biomarker Responses

Few of the biomarkers differed among sites despite much higher PFAS at the North region sites (Table 6 and Table 7). This indicates that PFAS in the North region, regardless of a 3- to 8-fold difference in exposure compared to the St. Louis River sites and being 10- to 25-fold higher than at the reference lake, were not causing noticeable physiological responses as measured by these particular biomarkers. This was similar to the results from Oscoda, MI where there was generally no difference in EROD, DNA-CV, plasma or glandular T4, or glandular T3 between the reference site and Clarks Marsh [21]. Oxidative stress and genotoxic effects have been noted in both invertebrates and fish [2], so either the biomarkers we measured in this study in tree swallows are not responsive to PFAS or the exposure was too low to elicit a response. The legacy organic contaminants such as PCBs, dioxins, and furans, which can induce EROD if exposure is sufficiently high, were also less than levels in the current study that have caused physiological responses in other tree swallow studies [11]. Finally, PFAS is apparently not clastogenic in birds as measured in this study by DNA-CV.

## 5. Conclusions

PFAS were elevated in tree swallow nestling eggs and carcasses at an AFFF source (ANG) and sites to the north that were connected hydrologically by surface and subsurface water. Sites along the St. Louis River were 10+ times lower than those sites influenced by the ANG site. There was also an AFFF source along the St. Louis River for which, while concentrations were slightly higher there than elsewhere on the St. Louis River, most likely the larger volume of water on the St. Louis River diluted the dietary contribution from that AFFF source. The PFHxS ratios and proportions of PFHxS to PFAS_40_ were evident at Boy Scout Landing, the AFFF site on the St. Louis River, even though total PFAS concentration at that site was not dissimilar to other locations along the river. The proportion of PFHxS to total PFAS, ratio of PFHxS/PFOS, and ratio of total carboxylic acids to total sulfonates, all seem to be good markers to distinguish the AFFF sources in the Duluth area from other PFAS sources, such as industrial and atmospheric sources. However, as noted earlier, these marker approaches may not work as well for the newer fire suppressing formulations, some of which do not contain PFAS, and those made using fluorotelomer processes. None of the physiological biomarker responses were associated with PFAS exposure even at the most highly PFAS-contaminated sites.

## Figures and Tables

**Table 1:** Detection frequencies as percentage of total samples for individual per- and polyfluoroalkyl substances (PFAS) detected in >50% of samples for PFAS_40_, when at least one matrix had >50% detections, in tree swallow tissues and diet from the Duluth, MN area, 2019–2021. Percentages >50% are in bold.

PFAS and Sample Size	Eggs	Nestling	Diet
Sample size (n)	47	83	9
Sulfonates			
PFOS	**100**	**99**	**100**
PFHxS	**96**	**86**	**67**
PFDS	**91**	**60** ^1^	22
Carboxylic acids			
PFOA	**72**	**90**	**89**
PFNA	**100**	**99**	**100**
PFDA	**98**	**88**	33
PFUnA	**98**	**71**	33
PFDoA	**100**	**52**	0
PFTrDA	**87**	19 ^1^	0
PFTeDA	**79**	19 ^1^	0
Other types			
N-EtFOSE	**64**	20 ^1^	0
7:3 FTCA	**60**	23 ^1^	11
6:2 FTS	13	17 ^1^	**56**
EtFOSAA	6	**56** ^1^	11

^1^ Sample size was 63 (2020 and 2021 data only) when these 7 analytes were also assessed. Note: perfluorobutanoate (PFBA), perfluoropentanoate (PFPeA), perfluorohexanoate (PFHxA), perfluoroheptanoate (PFHpA), perfluorooctanoate (PFOA), perfluorononanoate (PFNA), perfluorodecanoate (PFDA), perfluoroundecanoate (PFUnA), perfluorododecanoate (PFDoA), perfluorotridecanoate (PFTrDA), perfluorotetradecanoate (PFTeDA), perfluorobutanesulfonate (PFBS), perfluoropentanesulfonate (PFPeS), perfluorohexanesulfonate (PFHxS), perfluoroheptanesulfonate (PFHpS), perfluorooctanesulfonate (PFOS), perfluorononanesulfonate (PFNS), perfluorodecanesulfonate (PFDS), perfluorododecanesulfonate (PFDoS), perfluorooctanesulfonamide (PFOSA or FOSA), N-methylperfluorooctanesulfonamide, (N-MeFOSA), N-methylperfluorooctane-sulfonamidoacetic acid (N-MeFOSAA), N-ethylperfluorooctanesulfonamidoacetic acid (N-EtFOSAA), perfluoro(2-ethoxyethane) sulfonic acid (PFEESA), perfluoro-3-methoxypropanoic acid (PFMPA), perfluoro-3,6-dioxaheptanoic acid (PFMBA), 4:2 fluorotelomersulfonate (4:2 FTS), 6:2 fluorotelomersulfonate (6:2 FTS), 8:2 fluorotelomersulfonate (8:2 FTS), N-ethylperfluorooctanesulfonamide (N-EtFOSA), hexafluoropropylene oxide dimer acid (HFPO-DA), 4,8-dioxa-3H-perfuorononanoate (ADONA), 9-chlorohexadecafluoro-3-oxanonane-1-sulfonate (9Cl-PF3ONS), 11-chloroeicosafluoro-3-oxaundecane-1-sulfonate (11Cl-PF3OUdS), perfluoro-3-methoxypropanoic acid (PFMPA), and nonafluoro-3,6-dioxaheptanoic acid (NFDHA), 2H,2H,3H,3H-perfluorohexanoic acid (3:3 FTCA), 2H,2H,3H,3H-perfluorooctanoic acid (5:3 FTCA) and 2H,2H,3H,3H-perfluorodecanoic acid (7:3 FTCA), N-methyl perfluorooctane sulfonamidoethanol (N-MeFOSE), and N-ethyl perfluorooctane sulfonamidoethano (N-EtFOSE).

**Table 2:** Geometric means (ng/g wet wt.), 95% confidence intervals (CIs), and minimum and maximum values for individual per- and polyfluoroalkyl substances (PFAS) detected in greater than 50% of tree swallow egg samples from the Duluth, MN area, site 2020–2021.

	St. Louis River					North Region				Reference
PFAS	Thomson Reservoir	Boy Scout Landing	Munger Landing	Erie Ponds	Miller Creek	ANG	Martin Road	Rice Lake North	UMD	Boulder Lake
	n = 9	n = 6	n = 6	n = 3	n = 5	n = 4	n = 3	n = 3	n = 2	n = 5
Sulfonates										
PFOS	10.49 C ^1^ (7.60–14.48) 5.60–17.50	34.80 BC (14.49–83.57) 18.32–107	16.31 BC (11.45–23.24) 9.91–27.3	18.25 BC (14.50–22.97) 16.50–19.80	6.52 C (4.32–9.85) 4.38–8.67	136.90 AB (25.20–743) 29.50–339	92.08 AB (58.27–145.52) 81–114	469.7 A (28.25–7809) 181–1640	26.64 BC (8.29–85.57) 24.30–29.20	6.08 C (4.22–8.74) 3.93–8.24
PFHxS	0.23 CD (0.17–0.31) 0.13–0.39	1.52 BC (0.78–2.96) 0.73–3.55	0.39 CD (0.28–0.54) 0.26–0.61	0.50 CD (0.36–0.68) 0.44–0.56	0.26 CD (0.17–0.41) 0.15–0.39	11.00 AB (1.64–74.00) 2.60–42.8	4.47 AB (1.11–17.96) 2.95–8.45	31.74 A (0.85–1192) 12.90–171	1.14 BC (0.17–7.55) 0.98–1.32	0.12 D (0.07–0.21) 2ND-0.19
Carboxylic acids										
PFOA	0.25 BCD (0.12–0.53) 3ND ^2^-0.91	0.28 BCD (0.14–0.58) 1ND-0.55	0.17 CD (0.08–0.34) 3ND-0.43	0.12 CD (0.05–0.29) 2ND-0.18	0.10 D (0.08–0.13) 4ND-0.14	1.24 B (0.17–9.12) 0.20–3.06	0.44 BCD (0.15–1.31) 0.29–0.69	11.70 A (0.12–1142) 3.62–98.0	1.11 ABC (0.04–30.65) 0.85–1.44	0.37 BCD (0.21–0.66) 0.23–0.81
PFNA	1.50 BC (0.93–2.43) 0.71–4.26	1.40 BC (0.58–3.35) 0.58–6.28	1.03 BC (0.79–1.34) 0.77–1.59	0.51 C (0.37–0.7) 0.44–0.57	0.60 C (0.29–1.21) 0.29–1.39	2.63 AB (0.45–15.16) 0.51–5.29	0.82 BC (0.46–1.46) 0.63–0.97	11.03 A (0.33–363) 3.71–54.0	1.47 BC (0.23–9.37) 1.27–1.70	1.02 BC (0.72–1.44) 0.74–1.45
PFDA	0.81 A (0.56–1.18) 0.44–1.99	0.96 A (0.38–2.47) 0.37–5.18	0.96 A (0.47–1.94) 0.55–3.38	0.63 A (0.33–1.21) 0.47–0.74	0.62 A (0.45–0.86) 0.48–0.96	1.27 A (0.06–2)8.10 1ND-6.14	0.68 A (0.61–0.76) 0.66–0.72	2.39 A (0.14–41.4) 0.90–8.44	0.73 A (0.04–12.71) 0.58–0.91	0.64 A (0.44–0.93) 0.46–0.89
PFUnA	1.29 A (0.99–1.68) 0.76–2.31	1.51 A (0.59–3.87) 0.54–7.61	1.05 A (0.81–1.36) 0.80–1.59	0.68 A (0.43–1.07) 0.55–0.79	0.62 A (0.42–0.90) 0.42–0.80	1.12 A (0.06–20.50) 1 ND-3.49	0.98 A (0.93–1.05) 0.96–1.0	2.03 A (0.15–28.2) 0.77–6.28	0.89 A (0.59–1.34) 0.86–0.92	1.35 A (0.84–2.18) 0.91–2.2
PFDoA	0.61 AB ^3^ (0.46–0.81) 0.35–1.4	0.86 AB (0.30–2.47) 0.31–4.56	0.60 AB (0.46–0.78) 0.43–0.82	0.70 AB (0.44–1.12) 0.57–0.80	0.65 AB (0.36–1.18) 0.45–1.50	1.63 A (0.34–7.79) 0.49–5.48	0.65 AB (0.50–0.83) 0.61–0.73	0.88 AB (0.05–15.32) 0.34–3.17	0.71 AB (0.003–147.9) 0.47–1.08	0.51 B (0.30–0.88) 0.32–0.88
PFTrDA	0.47 A (0.20–1.06) 2ND-1.33	1.13 A (0.49–2.61) 0.44–4.33	0.33 A (0.10–1.14) 2ND-0.81	0.33 A (0.01–8.15) 1ND-0.70	0.63 A (0.40–1.01) 0.42–1.08	1.23 A (0.34–2.86) 0.60–1.71	0.75 A (0.58–0.99) 0.67–0.81	0.64 A (0.004–108.9) 1ND-4.49	0.82 A (0.02–31.4) 0.61–1.09	0.74 A (0.36–1.54) 0.37–1.33
PFTeDA	0.27 A (0.17–0.42) 1ND-0.58	0.29 A (0.09–0.94) 2ND-1.09	0.24 A (0.09–0.62) 2ND-0.63	0.37 A (0.17–0.84) 0.26–0.45	0.21 A (0.17–0.25) 0.17–0.25	0.61 A (0.07–5.62) 1ND-1.45	0.14 A (0.01–1.92) 2ND-0.48	0.35 A (0.005–27.24) 1ND-2.4	0.25 A (0.00001–519,704) 1ND-0.77	0.31 A (0.22–0.44) 0.25–0.43
Others										
PFDS	0.40 AB ^4^ (0.29–0.55) 0.23–0.98	0.47 AB (0.20–1.06) 0.18–1.87	0.36 AB (0.25–0.52) 0.26–0.68	1.57 A (0.55–4.49) 1.00–2.30	0.26 AB (0.15–0.46) 0.18–0.54	0.57 AB (0.04–9.01) 1ND-5.01	0.16 B (0.10–0.25) 0.13–0.18	0.19 AB (0.003–10.41) 2ND-1.21	0.28 AB (0.01–7.53) 0.22–0.37	0.20 AB (0.09–0.43) 1ND-0.40
N-EtFOSE	1.23 A (0.73–2.06) 2ND-3.32	2.22 A (0.55–8.96) 2ND-14	1.47 A (0.47–4.59) 3ND-7.27	0.71 A (0.30–1.65) 2ND-1.05	0.72 A (0.49–1.06) 3ND-1.11	1.75 A (0.22–13.70) 3ND-7.04	2.13 A (1.09–4.15) 1.5–2.66	2.19 A (0.03–147.7) 1ND-14.80	1.07 A (0.001–2383) 1ND-1.96	1.24 A (0.77–1.99) 0.85–2.07
7:3 FTCA	4.10 AB ^5^ (1.26–13.31) 5NA ^6^; 1ND-10.2	15.04 AB (7.73–29.26) 2NA; 10.82–27.6	4.04 B (0.70–23.29) 3NA; 1ND-8.32	19.95 AB (4.72–84.35) 11.80–37.20	7.57 AB (5.74–9.98) 5.62–10.10	25.32 AB (3.26–197) 7.27–154	8.78 AB (4.27–18.06) 1NA; 8.30–9.30	35.89 AB (0.00001–95,716,627) 1NA; 11.2–115	8.12 AB (5.28–12.49) 7.85–8.40	NA
Total PFAS_13_	15.51 BC (11.36–21.17) 8.71–24.97	42.46 BC (18.27–98.71) 21.07–134	20.58 BC (14.42–29.38) 12.87–35.68	21.36 BC (17.11–26.66) 19.34–23.00	9.51 C (6.77–13.36) 6.63–11.88	157.4 AB (28.20–879) 33.29–20.55	100.4 AB (61.21–164.64) 87.65–126	534.7 A (28.85–9909) 204–1981	32.74 BC (8.03–133.5) 29.31–36.57	10.13 C (6.85–14.97) 6.62–13.54
Total PFAS_40_	19.84 C (14.36–27.42) 10.8–32.62	57.97 BC (26.0–129.25) 24.39–183	27.57 C (17.63–43.11) 20.52–64.62	45.43 BC (20.79–99.28) 33.82–63.27	38.19 C (8.03–181.62) 17.51–355	208.2 AB (41.93–1034) 53.07–598	109.9 AB (65.46–184.56) 95.98–140	600.6 A (22.03–16,373) 206–2665	43.52 C (10.31–183.67) 38.86–48.74	13.71 C (8.83–21.26) 8.24–19.23
Percentage 13 of 40	78.2	73.2	74.6	47.0	24.9	75.6	91.3	89.0	75.2	73.9

^1^ Means sharing same letter are not significantly different among sites; ^2^ ND is number not detected; ^3^ alpha set to 0.12 to force Bonferroni separation among sites; ^4^ alpha set to 0.075 to force Bonferroni separation among sites; ^5^ alpha set to 0.25 to force Bonferroni separation among sites; ^6^ NA = number of samples in 2019 which were not analyzed for this PFAS. Note: perfluorooctanesulfonate (PFOS); perfluorohexanesulfonate (PFHxS); perfluorooctanoate (PFOA), perfluorononanoate (PFNA), perfluorodecanoate (PFDA), perfluoroundecanoate (PFUnA), perfluorododecanoate (PFDoA), perfluorotridecanoate (PFTrDA), perfluorotetradecanoate (PFTeDA), perfluorodecanesulfonate (PFDS), N-Ethylperfluorooctanesulfonamidoethanol (N-EtFOSE), 2H,2H,3H,3H-perfluorodecanoic acid (7:3 FTCA); Total PFAS_13_ is sum of 13 original PFAS analytes; Total PFAS_40_ is sum of 40 PFAS analytes. ANG = Duluth Air National Guard Base; UMD = farm at University of Minnesota—Duluth.

**Table 3:** Geometric means (ng/g wet wt.), 95% confidence intervals (CIs), and minimum and maximum values for individual per- and polyfluoroalkyl substances (PFAS) detected in greater than 50% of tree swallow nestling carcass samples from the Duluth, MN area, site 2019–2021.

PFAS	St. Louis River						North Region				Reference
	Thomson Reservoir	Boy Scout Landing	Munger Landing	Stryker Bay	Erie Ponds	Miller Creek	ANG	Martin Road	Rice Lake North	UMD	Boulder Lake
	n = 14	n = 6	n = 17	n = 5	n =14	n = 5	n = 5	n = 5	n = 6	n = 1	n = 5
Sulfonates											
PFOS	8.86 CD ^1^ (6.90–11.37) 3.29–14.30	12.76 BC (9.00–18.08) 8.52–18.60	11.63 BCD (9.97–13.56) 7.05–17.80	7.63 CD (5.53–10.53) 5.98–11.20	11.42 BCD (9.88–13.20) 8.44–19.20	6.08 CD (4.05–9.12) 4.08–9.80	36.73 AB (15.71–85.90) 17.6–109	82.10 A (44.89–150.1) 47.40–151	121.9 A (103.7–143.4) 104–155	3.72 D (.-.) 3.72	1.06 E (0.14–8.21) 1ND-2.53
PFHxS	0.28 CD (0.19–0.40) 5ND ^2^-0.49	1.29 B (0.74–2.25) 0.69–2.97	0.55 BC (0.44–0.69) 5ND-1.01	5ND	0.39 CD (0.29–0.53) 5ND-0.66	0.26 CD (0.20–0.33) 0.19–0.32	5.48 A (1.64–18.24) 1.54–21.6	7.00 A (5.10–9.61) 5.01–9.70	8.24 A (7.08–9.58) 6.93–10.5	0.24 CD (.-.) 0.24	0.17 D (0.07–0.42) 1ND-0.32
Carboxylic acids											
PFOA	1.08 ABC (0.79–1.48) 0.38–3.37	0.30 CDE (0.24–0.37) 0.23–0.41	0.59 BC (0.48–0.72) 0.26–1.04	0.48 BCD (0.25–0.93) 1ND-0.75	0.31 CDE (0.21–0.46) 3ND-0.71	0.09 E (0.02–0.37) 4ND-0.68	1.18 AB (0.41–3.40) 0.41–3.11	3.81 A (1.96–7.39) 2.00–6.40	3.88 A (3.06–4.91) 2.95–5.59	0.13 DE (.-.) 0.13	0.76 BC (0.54–1.06) 0.49–0.97
PFNA	2.05 A (1.66–2.52) 1.05–3.79	0.91 ABC (0.65–1.28) 0.63–1.47	1.12 AB (1.02–1.24) 0.79–1.69	0.85 ABC (0.59–1.22) 0.60–1.23	0.94 ABC (0.70–1.27) 0.42–2.80	0.44 C (0.19–0.98) 0.25–1.28	0.69 BC (0.46–1.05) 0.49–1.19	1.02 ABC (0.54–1.94) 0.64–2.03	1.73 A (1.28–2.32) 1.27–2.81	0.05 D (.-.) 1 ND	1.40 AB (1.10–1.78) 1.10–1.68
PFDA	0.53 AB (0.46–0.62) 0.32–0.75	0.33 AB (0.28–0.38) 0.25–0.38	0.42 AB (0.34–0.51) 3ND-0.67	0.23 B (0.15–0.36) 4ND-0.43	0.44 AB (0.34–0.56) 2ND-0.90	0.67 A (0.26–1.68) 0.31–2.15	0.33 AB (0.16–0.67) 0.21–0.91	0.39 AB (0.25–0.62) 0.25–0.62	0.76 A (0.59–0.98) 0.54–1.13	0.05 C (.-.) 1ND	0.34 AB (0.28–0.42) 0.29–0.40
PFUnA	0.57 A (0.47–0.71) 0.31–0.88	0.22 AB (0.10–0.47) 1ND-0.36	0.43 A (0.38–0.49) 1ND-0.70	0.31 AB (0.18–0.51) 2ND-0.45	0.30 AB (0.21–0.43) 4ND-0.59	0.08 BC (0.03–0.25) 4ND-0.42	0.08 BC (0.02–0.31) 4ND-0.55	0.05 C (.-.) 5ND	0.24 AB (0.07–0.83) 2ND-0.72	0.05 C (.-.) 1ND	0.42 A (0.34–0.53) 0.33–0.50
PFDoA	0.11 ABC (0.07–0.18) 8ND-0.21	0.08 BC (0.05–0.11) 3ND-0.13	0.16 ABC (0.15–0.18) 5ND-0.21	5ND	0.27 A (0.22–0.32) 5ND-0.48	0.17 AB (0.07–0.41) 1ND-0.36	0.12 ABC (0.03–0.44) 2ND-0.67	0.05 C (.-.) 5ND	0.06 BC (0.04–0.09) 5ND-0.13	0.05 C (.-.) 1ND	0.11 ABC (0.06–0.18) 1ND-0.15
Other types											
PFDS	0.17 B (0.10–0.29) 5NA; 2ND-0.38	0.09 BC (0.06–0.14) 2ND-0.13	0.18 B (0.16–0.19) 5NA; 0.13–0.21	NA	0.57 A (0.46–0.71) 5NA; 0.33–0.88	0.22 AB (0.14–0.35) 0.17–0.43	0.09 BC (0.02–0.33) 4ND-0.60	0.05 C (.-.) 5ND	0.05 C (.-.) 6ND	0.05 C (.-.) 1ND	0.05 C (.-.) 5ND
N-EtFOSAA	0.10 BC (0.05–0.18) 5NA ^3^; 5ND-0.48	0.18 B (0.13–0.26) 0.11–0.29	0.25 B (0.20–0.32) 5NA; 0.13–0.45	NA	1.08 A (0.89–1.31) 5NA; 0.75–1.63	0.23 B (0.08–0.67) 1ND-0.50	0.05 C (.-.) 5ND	0.05 C (.-.) 5ND	0.05 C (.-.) 6ND	0.05 C (.-.) 1ND	0.05 C (.-.) 5ND
Total PFAS_13_	13.57 C (10.81–17.04) 5.50–23.49	16.15 C (11.59–22.51) 10.8–23.6	15.06 C (13.07–17.35) 9.69–22.11	9.40 CD (6.62–13.34) 6.87–14.03	14.63 C (12.66–16.91) 10.01–25.87	8.06 CDE (5.06–12.84) 5.66–15.01	45.24 B (18.75–109.1) 20.65–139.4	94.82 AB (52.86–170.1) 55.29–169	138.23 A (117.5–162.6) 116–177	4.09 E (.-.) 4.09	5.04 DE (3.80–6.69) 3.66–6.49
Total PFAS_40_	14.26 DE (11.53–17.65) 5.50–23.49	16.53 DE (11.80–23.14) 10.97–24.27	17.35 DE (15.23–19.77) 12.38–32.10	9.40 EF (6.62–13.34) 6.87–14.03	20.57 DE (16.80–25.17) 10.01–39.74	30.88 CD (25.24–37.79) 25.72–39.65	54.64 BC (17.35–172.1) 20.82–249.7	96.37 AB (54.66–169.9) 58.49–170	139.5 A (118.9–163.8) 117–178	4.09 F (.-.) 4.09	5.58 F (4.24–7.35) 3.88–6.81
Percentage 13 is of 40	95.1	97.7	86.8	100	71.1	26.1	82.8	98.4	99.1	100	90.3

^1^ Means sharing same letter are not significantly different among sites; ^2^ ND is number not detected; ^3^ NA = number of samples in 2019 which were not analyzed for this PFAS. Note: perfluorooctanesulfonate (PFOS); perfluorohexanesulfonate (PFHxS); perfluorooctanoate (PFOA), perfluorononanoate (PFNA), perfluorodecanoate (PFDA), perfluoroundecanoate (PFUnA),), perfluorododecanoate (PFDoA), perfluorodecanesulfonate (PFDS), N-ethylperfluorooctanesulfonamidoacetic acid (N-EtFOSAA); Total PFAS_13_ is sum of 13 original PFAS analytes; and Total PFAS_40_ is sum of 40 PFAS analytes.

**Table 4:** Average accumulation rate (ng/day) of perfluorohexane sulfonate (PFHxS) and perfluorooctane sulfonate (PFOS) in tree swallow nestling carcasses from the Duluth, MN area in 2020–2021.

Site		PFHxS	PFOS
	n	ng/d	ng/d
Reference			
Boulder Lake	5	0.34 CD ^1^	2.70 D
North region			
Rice Lake North	6	8.25 A	134.80 A
Martin Road	5	11.66 A	130.98 A
ANG	5	7.27 A	39.53 B
UMD Farm	1	0.16 D	1.19 D
St. Louis River			
Thomson Reservoir	9	0.43 CD	11.72 C
Boy Scout Landing	5	1.72 B	14.35 BC
Munger Landing	12	0.89 BC	17.16 BC
Erie Ponds	9	0.54 BC	14.42 BC
Miller Creek	5	0.38 CD	8.99 C

^1^ Means sharing same letter are not significantly different; 1-way ANOVA, *p* < 0.001 for the two PFAS individually.

**Table 5:** Concentrations of per- and polyfluoroalkyl substances (PFAS) in composited tree swallow diet samples (ng/g wet wt.) from the Duluth, MN area, in 2020 and 2021. There was one composited sample per site per year.

PFAS	St. Louis River				North Region			Reference
	Thomson Reservoir	Boy Scout Landing	Erie Ponds	Miller Creek	ANG	Martin Road	Rice Lake North	Boulder Lake
Sulfonates								
PFOS	15.5	5.10	4.86	2.92	29.0	44.7	90.0	2.95
PFHxS	0.29	0.31	.^1^	.	3.01	0.54	1.73	.
Carboxylic acids								
PFOA	0.86	0.12	0.7	0.22	0.94	1.13	1.50	0.451
PFNA	2.84	0.39	0.46	0.25	0.37	0.68	1.33	1.51
PFDA	0.61	.	.	.	.	.	0.47	0.322
PFUnA	0.55	.	.	.	.	.	0.27	0.283
6:2 FTS	.	2.24	1.91	1.74	.	43.9	1.02	.
8:2 FTS	.	.	152	.	0.65	1.03	0.72	.
Total PFAS_13_	20.6	5.92	6.02	3.32	33.3	47.0	95.30	5.52
Total PFAS_40_	20.6	8.15	160	5.13	34.0	92.0	97.0	5.52
Percentage 13 is of 40	100	72.6	3.8	66.1	98.1	51.1	98.2	100

^1^ Dot are samples below the detection limit. Note: perfluorooctanesulfonate (PFOS); perfluorohexanesulfonate (PFHxS); perfluorooctanoate (PFOA), perfluorononanoate (PFNA), perfluorodecanoate (PFDA), perfluoroundecanoate (PFUnA), 6:2 fluorotelomersulfonate (6:2 FTS), 8:2 fluorotelomersulfonate (8:2 FTS); Total PFAS_13_ is sum of 13 original PFAS analytes; Total PFAS_40_ is sum of 40 PFAS analytes.

**Table 6:** Oxidative stress responses (means [μmol/g of tissue], 95% confidence intervals in parentheses, and minimum–maximum values) in tree swallow nestling liver samples, from the Duluth, MN area by year and location 2019–2021.

Site and Year	N	Thiol	PBSH	Total GSH	Reduced GSH	GSSG	GSSG_GSH	TBARS Protein	TBARS Tissue
Reference									
Boulder Lake 2021	6	38.17 AB ^1^ (27.43–48.91) 25.5–51.2	28.35 ABC (20.14–36.56) 18.3–38.2	16.56 AB (13.57–19.55) 13.5–20.2	9.82 AB ^2^ (7.13–12.50) 7.2–13.0	3.37 A (3.18–3.56) 3.2–3.6	0.36 A (0.28–0.44) 0.27–0.44	550.88 AB (229.84–871.92) 169.0–936.5	64.1 AB (26.28–101.95) 20.0–118.1
North region									
Rice Lake North 2021	5	39.70 AB (29.94–49.46) 29.6–46.8	28.88 ABC (20.89–36.87) 21.8–36.6	17.81 A (15.37–20.25) 14.6–19.7	10.82 A (8.11–13.53) 7.8–13.3	3.49 A (2.99–4.00) 3.1–4.2	0.34 A (0.23–0.45) 0.24–0.44	327.52 AB (231.11–423.93) 246.6–436.7	43.68 AB (28.30–59.06) 28.2–60.9
Martin Road 2021	4	31.85 AB (20.34–43.36) 24.8–40.5	23.28 ABC (13.61–32.94) 17.5–29.2	15.06 AB (10.55–19.56) 12.1–18.6	8.59 AB (5.55–11.62) 7.3–11.3	3.24 A (2.30–4.17) 2.4–3.7	0.38 A (0.28–0.48) 0.32–0.44	428.1 AB (220.04–636.16) 289.0–603.3	49.38 AB (23.64–75.11) 30.0–69.2
ANG 2021	5	31.24 AB (26.19–36.29) 25.3–35.4	22.52 ABC (20.22–24.82) 19.8–24.9	14.44 AB (10.43–18.44) 10.9–18.0	8.75 AB (5.87–11.62) 5.6–10.9	2.85 A (2.07–3.63) 2.2–3.6	0.34 A (0.23–0.44) 0.23–0.47	318.76 AB (151.54–485.98) 177.2–485.5	32.52 AB (10.95–54.09) 15.0–59.0
UMD Farm 2021	1	24.00 B	18.7 C	9.57 B	5.27 B	2.15 A	0.41 A	115.4 B	11.30 B
St. Louis River									
Thomson Reservoir 2019	10	45.35 AB (36.17–54.53) 25.6–66.1	36.61 ABC (28.94–44.28) 21.6–57.6	14.77 AB (11.66–17.88) 6.7–22.3	8.76 AB (6.66–10.86) 4.00–14.3	3.02 A (2.42–3.62) 1.40–4.00	0.36 A (0.31–0.41) 0.26–0.46	707.94 AB (499.09–916.79) 431.2–1308.4	84.67 AB (57.66–111.68) 53.50–173.1
Thomson Reservoir 2021	10	30.23 AB (22.22–38.24) 15.8–53.7	22.09 ABC (15.81–28.37) 12.4–41.0	14.56 AB (11.57–17.54) 8.3–22.4	8.14 AB (6.16–10.12) 3.4–12.6	3.21 A (2.65–3.77) 2.2–4.9	0.42 A (0.33–0.51) 0.31–0.74	830.66 A (597.76–1063.6) 304.7–1357	92.00 A (68.14–115.86) 33.8–157.2
Boy Scout Landing 2021	4	28.20 AB (6.72–49.68) 17.8–48.0	20.63 BC (2.66–38.59) 13.8–37.4	13.80 AB (6.60–21.01) 7.5–17.8	7.60 AB (3.29–11.91) 4.1–10.6	3.10 A (1.55–4.66) 1.7–3.9	0.415 A (0.33–0.50) 0.34–0.47	276.7 AB (131.59–421.81) 190.3–375.0	32.23 AB (14.81–49.64) 22.6–46.9
Munger Landing 2019	5	44.86 AB (27.20–62.52) 24.7–64.4	36.14 ABC (21.56–50.72) 20.5–53.2	14.52 AB (9.74–19.30) 8.4–19.0	8.72 AB (5.40–12.04) 4.2–11.2	2.88 A (2.06–3.70) 2.1–3.9	0.348 A (0.24–0.45) 0.28–0.49	575.42 AB (362.21–788.63) 416.7–856.6	59.38 AB (42.42–76.34) 41.7–76.3
Munger Landing 2021	7	29.27 AB (20.31–38.23) 18.3–46.8	21.31 ABC (13.91–28.72) 11.0–34.9	14.6 AB (11.18–18.02) 9.92–20.36	7.95 AB (5.63–10.26) 4.36–11.94	3.32 A (2.68–3.96) 2.11–4.21	0.44 A (0.34–0.54) 0.35–0.64	634.49 AB (464.50–804.47) 267.5–840.4	70.76 AB (47.66–93.86) 33.3–113.8
Stryker Bay 2019	10	47.85 AB (41.03–54.67) 35.2–65.6	38.12 AB (32.46–43.78) 28.4–52.9	16.90 AB (14.46–19.34) 10.9–21.1	9.73 AB (8.34–11.12) 6.8–12.7	3.58 A (2.88–4.28) 2.0–4.8	0.3 A (0.31–0.43) 0.28–0.53	477.24 AB (294.52–659.96) 204.4–988.8	58.29 AB (35.59–80.99) 23.1–125.9
Erie Ponds 2019	9	50.28 A (43.27–57.28) 37.8–62.7	40.544 A (34.84–46.25) 30.7–51.1	17.17 AB (15.14–19.19) 13.4–19.8	9.73 AB (8.27–11.20) 7.1–12.0	3.70 A (3.20–4.20) 2.6–4.5	0.39 A (0.33–0.45) 0.29–0.49	653.21 AB (571.09–735.33) 477.1–871.6	73.97 AB (60.87–87.06) 48.4–112.0
Erie Ponds 2021	10	36.74 AB (30.06–43.42) 24.3–49.1	26.25 ABC (21.16–31.34) 16.4–38.0	16.87 AB (14.65–19.09) 13.1–22.1	10.49 AB (8.57–12.41) 7.1–14.7	3.19 A (2.83–3.55) 2.5–3.8	0.32 A (0.26–0.38) 0.20–0.47	631.69 AB (400.82–862.56) 211.5–1257.4	71.17 AB (45.42–96.92) 27.2–143.0
Miller Creek 2021	10	24.21 B (17.79–30.63) 14.4–42.6	17.68 C (12.81–22.55) 10.1–31.6	12.23 AB (10.13–14.33) 8.5–17.5	6.55 AB (4.95–8.14) 4.02–11.0	2.85 A (2.55–3.14) 2.1–3.4	0.46 A (0.39–0.53) 0.30–0.59	720.55 AB (413.86–1027.2) 275.8–1549.7	83.3 AB (48.09–118.51) 29.7–187.2
*p*-value		<0.0001	<0.0001	0.0584	0.0386	0.2027	0.0780	0.0035	0.0074

Note: protein bound sulfhydryl (PBSH); reduced glutathione (GSH); oxidized glutathione (GSSG); thiobarbituric acid reactive substances (TBARS); total sulfhydryl (TS). ^1^ Means sharing same letter are not significantly different among sites. ^2^ alpha set to 0.1 to force Bonferroni mean separations for reduced GSH.

**Table 7:** Mean, 95% confidence intervals in parentheses, and minimum–maximum values for immune responses (μg/mL), thyroid hormone concentrations (ng/mL plasma, ng/mg gland), ethoxyresorufin-O-dealkylase (EROD, pmol of product/min/mg microsomal protein) activity, and DNA coefficient of variation (CV) in tree swallow nestlings in the Duluth, MN area 2019–2021.

Site and Year		Immune Responses		Thyroid Levels				EROD	Percent CV
	N	PIT54	IgY	T3 Plasma	T4 Plasma	T3 Thyroid gland	T4 Thyroid gland		
Reference									
Boulder Lake 2021	6	0.17 A ^1^ (0.14–0.20) 0.11–0.20	111.17 A (75.68–146.65) 86.9–174.2	NA ^2^	NA	2.63 AB (2.05–3.21) 2.0–3.5	318.75 ABC (208.8–428.7) 188.1–459.7	17.78 B (11.16–24.39) 11.8–28.6	1.78 A (0.81–2.75) 0.4–2.8
North region									
Rice Lake North 2021	5	0.14 A (0.12–0.16). 0.13–0.16	76.40 A (47.95–104.85) 41.3–96.9	NA	NA	3.30 A (2.76–3.84) 2.8–3.8	346.98 AB (271.2–422.8) 296.9–447.0	18.49 B (11.30–25.69) 8.75–23.38	2.52 A (1.60–3.44) 1.5–3.3
Martin Road 2021	4	0.13 A (0.08–0.19) 0.08–0.16	61.49 A (19.61–103.36) 33.9–91.4	NA	NA	1.68 AB (1.15–2.20) 1.3–2.0	225.58 ABCD (41.29–409.9) 59.6–317.0	9.79 B (4.79–14.79) 5.7–12.4	2.45 A (1.33–3.57) 1.7–3.1
ANG 2021	5	0.15 A (0.12–0.17) 0.12–0.18	103.06 A (61.40–144.72) 69.9–153.2	NA	NA	1.34 AB (0.241–2.44) 0.8–2.9	120.8 CD (0.257–241.3) 32.8–271.5	17.62 B (-1.39–36.63) 7.47–43.96	2.20 A (1.40–3.00) 1.5–3.0
UMD Farm 2021	1	NA	48.6 A	NA	NA	0.9 B	42.1 D	5.05 B	3.5 A
St. Louis River									
Thomson Reservoir 2019	10	NA	NA	0.60 A (0.38–0.81) 0.3–1.2	13.17 A (10.55–15.79) 7.5–19.7	3.04 A (2.66–3.43) 2.2–3.7	403.82 A (352.97–454.67) 273.8–494.6	31.66 AB (16.35–46.96) 8.6–69.7	2.36 A (1.62–3.10) 0.8–3.5
Thomson Reservoir 2021	10	0.13 A (0.11–0.15) 0.09–0.17	61.17 A (50.11–72.23) 39.0–85.9	NA	NA	2.57 AB (1.62–3.52) 1.5–6.0	357.9 AB (253.9–461.9) 177.1–651.8	13.50 B (9.25–17.75) 6.1–22.6	2.06 A (1.25–2.87) 0.6–3.9
Boy Scout Landing 2021	4	0.13 A (0.11–0.15) 0.11–0.14	72.66 A (33.02–112.30) 55.0–109.0	NA	NA	2.23 AB (1.61–2.84) 2.0–2.8	256.2 ABC (213.6–298.7) 221.3–279.9	12.54 B (5.21–19.86) 8.5–9.1	2.03 A (0.98–3.07) 1.2–2.8
Munger Landing 2019	5	NA	NA	0.572 A (0.34–0.81) 0.42–0.9	13.06 A (10.79–15.33) 10.4–15	2.844 AB (0.57–5.12) 1.23–4.98	222.8 ABCD (97.27–348.33) 120.4–349.2	98.50 A (-11.88–208.9) 27.72–243.41	3.06 A (1.40–4.72) 1.1–4.6
Munger Landing 2021	7	0.152 A (0.12–0.19) 0.091–0.199	76.132 A (49.44–102.83) 37.7–125.8	NA	NA	2.157 AB (1.70–2.61) 1.5–3	276.7 ABC (201.3–352.0) 215.6–439.8	21.622 B (13.81–29.43) 8.14–32.21	2.014 A (1.26–2.77) 1.1–3.5
Stryker Bay 2019	10	NA	NA	0.557 A (0.45–0.66) 0.3–0.78	15.58 A (13.12–18.04) 9.2–20.1	2.715 AB (2.05–3.38) 1.56–4.23	199.69 ABCD (134.45–264.93) 126.8–434.5	35.447 AB (26.73–44.16) 10.49–53.52	3.03 A (2.44–3.62) 1.6–4
Erie Ponds 2019	9	NA	NA	0.47 A (0.37–0.57) 0.33–0.68	11.411 A (9.35–13.48) 7.9–16	2.226 AB (1.86–2.60) 1.44–2.78	181.066 BCD (132.85–229.29) 81.5–286.2	62.638 AB (17.91–107.37) 10.75–186.59	2.3 A (1.65–2.95) 1.4–4.1
Erie Ponds 2021	10	0.145 A (0.13–0.16) 0.112–0.193	88.105 A (63.44–112.77) 38.4–134.7	NA	NA	2.57 AB (1.91–3.23) 1.7–4.7	296.76 ABC (263.0–330.6) 233.5–385.3	37.083 AB (30.65–43.52) 21.18–50.11	2.38 A (1.73–3.03) 0.8–3.4
Miller Creek 2021	10	0.119 A (0.10–0.14) 0.068–0.179	86.445 A (57.24–115.65) 34.45–188.8	NA	NA	2.25 AB (1.81–2.69) 1.4–3.6	139.64 CD (105.8–173.5) 80.1–215.3	27.182 B (18.43–35.94) 11.42–58.68	2.5 A (1.48–3.52) 0.5–5.3
*p*-value		0.1193	0.0807	0.6068	0.0555	0.0128	<0.0001	<0.0001	0.4584

^1^ Means sharing same letter are not significantly different among site-year combinations; ^2^ NA = not analyzed. Note: Haptoglobin-like activity (PIT54); immunoglobulin (IgY); Triiodothyronine (T3); thyroxine (T4); Ethoxyresorufin-O-dealkylase (EROD); coefficient of variation (CV).

## Data Availability

All data collected for this study are available at https://doi.org/10.5066/P149HMI2 (Custer, C.M. 2024).

## Supplementary Materials

The following supporting information can be downloaded at: https://www.mdpi.com/article/10.3390/toxics12090660/s1, Figure S1: Correlation between the sum of the original 13 per- and polyfluoroalkyl substances (PFAS_13_) and the sum of the current 40 PFAS (PFAS_40_) in tree swallow nestlings from the Duluth, MN area in 2021. Note: ANG = Duluth Air National Guard Base. Figure S2: Geometric means (bars, ng/g wet wt.) and 95% confidence intervals (vertical lines) for perfluorooctane sulfonate (PFOS, upper panel) and perfluorohexane sulfonate (PFHxS, lower panel) in tree swallow eggs (left side) and nestling carcasses (right side) from the Duluth, MN area, 2019–2021. Means sharing same letter are not significantly different, 1-way analysis of variance (ANOVA). Figure S3: Geometric means (bars, ng/g wet wt.) and 95% confidence intervals (vertical lines) for perfluorooctanoate (PFOA, upper panel) and perfluorononanoate (PFNA, lower panel) in tree swallow eggs (left side) and nestling carcasses (right side) from the Duluth, MN area, 2019–2021. Means sharing same letter are not significantly different, 1-way analysis of variance (ANOVA). Figure S4: Nonmetric multidimensional scaling (NMDS) plot of per- and polyfluoroalkyl substances (PFAS) in tree swallow egg samples (2020 and 2021) in the Duluth, MN area separated by region for n = 21 PFAS detected in >2 samples. Note that NMDS plots are unitless. Figure S5: Nonmetric multidimensional scaling (NMDS) plot of per- and polyfluoroalkyl substances (PFAS) by individual site in tree swallow nestling samples (2010 and 2021) in the Duluth, MN area for n = 25 PFAS detected in >2 samples. Note that NMDS plots are unitless. Figure S6: Nonmetric multidimensional scaling (NMDS) plot of per- and polyfluoroalkyl substances (PFAS) by individual site in tree swallow egg samples (2010 and 2021) in the Duluth, MN area for n = 21 PFAS detected in >2 samples. Note that NMDS plots are unitless. Table S1: Geometric mean detection limits (DL, ng/g wet wt.), number of samples above the detection limit, and range of detection limits by matrix and year individually for per- and polyfluoroalkyl substances (PFAS) in tree swallow tissues from the Duluth, MN area in 2019–2021. Table S2: Average percent recovery for 40 per- and polyfluoroalkyl substances (PFAS) in tree swallow tissues from the Duluth, MN areas, 2020–2021. Table S3: Detection limits for organochlorine contaminants in tree swallows from the Duluth, MN area in 2019–2021. Table S4: Detection limits, number and percent of samples above the detection limit, and percent recovery for elements in tree swallow liver tissue from the Duluth, MN area between 2019–2021. Table S5: Geometric mean concentrations (ng/g wet wt., except as note below) and 95% confidence intervals (CIs) for other chemical contaminants in tree swallow nestling carcasses at sites in the Duluth, MN area in 2020–2021. Table S6: Geometric means (ng/g wet wt.), 95% confidence intervals (CIs), minimum and maximum values for contaminants detected in greater than 50% of tree swallow egg samples from Boulder Lake, north of Duluth, MN in 2020. Table S7: Geometric mean concentrations (µg/g dry wt.), 95% confidence intervals (CIs), and minimum and maximum values for elements detected in >50% of tree swallow nestling liver samples collected in the Duluth, MN area, 2019–2021.
