# Supplementary material for: Per- and Polyfluoroalkyl Substances in the Duluth, Minnesota Area: Exposure to and Biomarker Responses in Tree Swallows Relative to Known Fire-Fighting Foam Sources
Source: Toxics. 2024 Sep 10;12(9):660. doi: 10.3390/toxics12090660 (PMC11435444; doi:10.3390/toxics12090660)
Supplement: Supplementary file 1 [file toxics-12-00660-s001.zip › toxics-3144149-supplementary.pdf]

Supplemental Table S1. Geometric mean detection limits (DL, ng/g wet wt.), number of samples above the detection limit, and range of detection limits by matrix and year individually for per- and polyfluoroalkyl substances in tree swallow tissues from the Duluth, MN area in 2019 -2021.

PFAS	Eggs				Nestling carcasses						Diet			
name	2020		2021		2019		2020		2021		2020		2021	
	n=17		n=30		n=20		n=19		n=44		n=3		n=6	
	Geomean	# > DL	Geomean	# > DL	Geomean	# > DL	Geomean	# > DL	Geomean	# > DL	Geomean	# > DL	Geomean	# > DL
PFBA	0.49	0	1.00	0	1.55	0	0.47	0	0.43	6	1.26	0	0.41	0
PFPeA	0.24	0	0.43	0	0.78	0	0.23	1	0.22	5	0.63	1	0.20	0
PFHxA	0.12	0	0.21	0	0.39	0	0.14	2	0.15	9	0.58	0	0.11	0
PFHpA	0.12	1	0.18	0	0.39	0	0.12	2	0.11	5	0.31	0	0.10	1
PFOA	0.12	17	0.24	17	0.39	17	0.12	19	0.11	39	0.31	2	0.10	6
PFNA	0.12	17	0.19	30	0.39	20	0.12	19	0.10	43	0.31	3	0.10	6
PFDA	0.12	17	0.16	29	0.39	11	0.12	19	0.10	43	0.31	2	0.10	1
PFUnA	0.12	17	0.16	29	0.39	14	0.12	18	0.10	27	0.31	2	0.10	1
PFDoA	0.12	17	0.16	30	0.39	1	0.12	13	0.10	29	0.31	0	0.10	0
PFTrDA	0.12	12	0.16	29	NA[1]	NA	0.12	10	0.10	2	0.31	0	0.10	0
PFTeDA	0.12	17	0.18	20	NA	NA	0.12	2	0.10	10	0.31	0	0.10	0
PFBS	0.12	0	0.16	0	0.39	0	0.12	0	0.10	0	0.31	0	0.10	1
PFPeS	0.12	0	0.16	5	NA	NA	0.12	0	0.11	8	0.31	0	0.10	1
PFHxS	0.12	15	0.16	30	0.39	9	0.12	18	0.10	44	0.31	2	0.10	4
PFHpS	0.12	5	0.16	15	NA	NA	0.12	6	0.10	17	0.31	1	0.10	1
PFOS	0.12	17	0.16	30	0.39	20	0.12	18	0.11	44	0.31	3	0.11	6
PFNS	0.12	1	0.16	2	NA	NA	0.12	0	0.10	1	0.31	0	0.10	0
PFDS	0.12	15	0.16	28	NA	NA	0.12	10	0.10	28	0.31	1	0.10	1
PFDoS	0.12	0	0.16	1	NA	NA	0.12	0	0.10	0	0.31	0	0.10	0
4:2 FTS	0.49	0	0.65	1	NA	NA	0.47	0	0.41	0	1.26	0	0.41	0
6:2 FTS	0.44	3	0.59	3	NA	NA	0.42	1	0.37	10	1.13	0	0.37	5
8:2 FTS	0.49	0	0.65	3	NA	NA	0.47	0	0.43	1	1.26	0	0.41	4
PFOSA	0.12	0	0.16	2	0.39	5	0.12	9	0.10	12	0.31	0	0.10	2
N-MeFOSA	0.14	0	0.19	0	NA	NA	0.14	1	0.12	3	0.36	0	0.12	0
N-EtFOSA	0.30	0	0.41	0	NA	NA	0.30	0	0.26	0	0.79	0	0.25	0
MeFOSA	0.12	2	0.16	3	NA	NA	0.12	1	0.10	22	0.31	0	0.10	0
EtFOSAA	0.12	0	0.16	3	NA	NA	0.12	8	0.10	27	0.31	0	0.10	1

N-MeFOSE_	1.22	0	1.63	0	NA	NA	1.17	0	1.02	0	3.15	0	1.02	0
N-EtFOSE	0.91	15	1.39	15	NA	NA	1.00	2	0.76	15	2.36	0	0.76	2
HFPO-DA	0.46	0	0.62	0	NA	NA	0.44	0	0.39	0	1.20	0	0.39	0
ADONA	0.49	0	0.65	0	NA	NA	0.47	0	0.41	0	1.26	0	0.41	0
9Cl-PF3ONS	0.49	0	0.65	0	NA	NA	0.47	0	0.41	0	1.26	0	0.41	0
11Cl-PF3OUdS	0.49	0	0.65	0	NA	NA	0.47	0	0.41	0	1.26	0	0.41	0
3:3 FTCA	NA	NA	0.77	0	NA	NA	NA	NA	0.41	0	NA	NA	0.41	0
5:3 FTCA	NA	NA	4.06	4	NA	NA	NA	NA	2.90	7	NA	NA	2.55	0
7:3 FTCA	NA	NA	4.06	28	NA	NA	NA	NA	2.54	19	NA	NA	2.55	1
PFEESA	NA	NA	0.16	0	NA	NA	NA	NA	0.10	0	NA	NA	0.10	0
PFMPA	NA	NA	0.33	0	NA	NA	NA	NA	0.20	0	NA	NA	0.20	0
PFMBA	NA	NA	0.16	1	NA	NA	NA	NA	0.10	10	NA	NA	0.10	0
NFDHA	NA	NA	0.27	0	NA	NA	NA	NA	0.32	0	NA	NA	0.20	0

[1] NA = analyte not analyzed that year

Note: text in red are analytes with 0 detections across all years and matrix types

Note: perfluorobutanoate (PFBA), perfluoropentanoate (PFPeA), perfluorohexanoate (PFHxA), perfluoroheptanoate (PFHpA), perfluorooctanoate (PFOA), perfluorononanoate (PFNA), perfluorodecanoate (PFDA), perfluoroundecanoate (PFUnA), perfluorododecanoate (PFDoA), perfluorotridecanoate (PFTrDA), perfluorotetradecanoate (PFTeDA), perfluorobutanesulfonate (PFBS), perfluoropentanesulfonate (PFPeS), perfluorohexanesulfonate (PFHxS), perfluoroheptanesulfonate (PFHpS), perfluorooctanesulfonate (PFOS), perfluorononanesulfonate (PFNS), perfluorodecanesulfonate (PFDS), perfluorododecanesulfonate (PFDoS), perfluorooctanesulfonamide (PFOSA or FOSA), N-methylperfluorooctanesulfonamide, (N-MeFOSA), N-methylperfluor-1-ooctane-sulfonamidoacetic acid (N-MeFOSAA), N-ethylperfluoro-1-octanesulfonamidoacetic acid (N-EtFOSAA), perfluoro(2-ethoxyethane) sulfonic acid (PFEESA), perfluoro-3-methoxypropanoic acid (PFMPA), perfluoro-3,6-dioxaheptanoic acid (PFMBA), 4:2 fluorotelomersulfonate (4:2 FTS), 6:2 fluorotelomersulfonate (6:2 FTS), 8:2 fluorotelomersulfonate (8:2 FTS), N-ethylperfluorooctanesulfonamide (N-EtFOSA), hexafluoropropylene oxide dimer acid (HFPO-DA), 4,8-dioxa-3H-perfuorononanoate (ADONA), 9-chlorohexadecafluoro-3-oxanonane-1-sulfonate (9Cl-PF3ONS), 11-chloroeicosafluoro-3-oxaundecane-1-sulfonate (11Cl-PF3OUdS), perfluoro-3-methoxypropanoic acid (PFMPA), and nonafluoro-3,6-dioxaheptanoic acid (NFDHA), 2H,2H,3H,3H-perfluorohexanoic acid (3:3 FTCA), 2H,2H,3H,3H-perfluorooctanoic acid (5:3 FTCA) and 2H,2H,3H,3H-perfluorodecanoic acid (7:3 FTCA), N-methyl perfluoro-1-octane sulfonamidoethanol (N-MeFOSE), and N-ethyl perfluoro-1-octane sulfonamidoethano (N-EtFOSE)

Supplemental Table S2. Average percent recovery for 40 per- and polyfluoroalkyl substances (PFAS) in tree swallow tissues from the Duluth, MN areas, 2020-2021.

PFAS name	Average % Recovery	PFAS continued	Average % Recovery
PFBA	100.6		
PFPeA	99.7	8:2 FTS[1]	140.5
PFHxA	106.4	N-MeFOSA[1]	105.4
PFHpA	96.8	N-EtFOSA[1]	101.6
PFOA	96.9	MeFOSAA[1]	102.9
PFNA	103.8	EtFOSAA[1]	101.6
PFDA	102.3	N-MeFOSE[1]	184.6
PFUnA	109.7	N-EtFOSE[1]	111.3
PFDoA	104.8	HFPO-DA[1]	99.2
PFTrDA[1]	118.4	ADONA[1]	113.6
PFTeDA[1]	110.8	9Cl-PF3ONS[1]	107.6
PFBS	99.4	11Cl-PF3OUdS[1]	100.3
PFPeS[1]	105.7	3:3 FTCA[2]	76.9
PFHpS[1]	96.0	5:3 FTCA[2]	128.0
PFHxS	101.6	7:3 FTCA[2]	114.0
PFOS	102.5	PFEESA[2]	96.1
PFNS[1]	92.9	PFMPA[2]	94.7
PFDS[1]	89.2	PFMBA[2]	94.6
PFDoS[1]	59.2	NFDHA[2]	77.1
PFOSA	103.4		
4:2 FTS[1]	105.1		
6:2 FTS[1]	121.1		

[1] analyzed beginning in 2020
[2] analyzed beginning in 2021

Note: perfluorobutanoate (PFBA), perfluoropentanoate (PFPeA), perfluorohexanoate (PFHxA), perfluoroheptanoate (PFHpA), perfluorooctanoate (PFOA), perfluorononanoate (PFNA), perfluorodecanoate (PFDA), perfluoroundecanoate (PFUnA), perfluorododecanoate (PFDoA), perfluorotridecanoate (PFTrDA), perfluorotetradecanoate (PFTeDA), perfluorobutanesulfonate (PFBS), perfluoropentanesulfonate (PFPeS), perfluorohexanesulfonate (PFHxS), perfluoroheptanesulfonate (PFHpS), perfluorooctanesulfonate (PFOS), perfluorononanesulfonate (PFNS), perfluorodecanesulfonate (PFDS), perfluorododecanesulfonate (PFDoS), perfluorooctanesulfonamide (PFOSA or FOSA), N-methylperfluorooctanesulfonamide, (N-MeFOSA), N-methylperfluoro-1-octane-sulfonamidoacetic acid (N-MeFOSAA), N-ethylperfluoro-1-octanesulfonamidoacetic acid (N-EtFOSAA), perfluoro(2-ethoxyethane) sulfonic acid (PFEESA), perfluoro-3-methoxypropanoic acid (PFMPA), perfluoro-3,6-dioxaheptanoic acid (PFMBA), 4:2 fluorotelomersulfonate (4:2 FTS), 6:2 fluorotelomersulfonate (6:2 FTS), 8:2 fluorotelomersulfonate (8:2 FTS), N-ethylperfluorooctanesulfonamide (N-EtFOSA), hexafluoropropylene oxide dimer acid (HFPO-DA), 4,8-dioxa-3H-perfuorononanoate (ADONA), 9-chlorohexadecafluoro-3-oxanonane-1-sulfonate (9Cl-PF3ONS), 11-chloroeicosafluoro-3-oxaundecane-1-sulfonate (11Cl-PF3OUdS), perfluoro-3-methoxypropanoic acid (PFMPA), and nonafluoro-3,6-dioxaheptanoic acid (NFDHA), 2H,2H,3H,3H-perfluorohexanoic acid (3:3 FTCA), 2H,2H,3H,3H-perfluorooctanoic acid (5:3 FTCA) and 2H,2H,3H,3H-perfluorodecanoic acid (7:3 FTCA), N-methyl perfluoro-1-octanesulfonamidoethanol (N-MeFOSE), and N-ethyl perfluoro-1-octanesulfonamidoethano (N-EtFOSE)

Supplemental Table S3. Detection limits for organochlorine contaminants in tree swallows from the Duluth, MN area in 2019-2021.

Contaminant	Detection limit (ng/g)
2,4'-DDE	0.04
4,4'-DDE	0.06
2,4'-DDD	0.05
4,4'-DDD	0.06
2,4'-DDT	0.06
4,4'-DDT	0.06
Aldrin	0.25
Benzene, Dichloro	0.40
Benzene, Trichloro	0.33
Benzene, Tetrachloro	0.12
Benzene, Pentachloro	0.17
Benzene, Hexachloro	0.09
Chlordane, gamma (trans)	0.08
Chlordane, alpha (cis)	0.09
Chlordane, oxy-	0.72
Dieldrin	0.17
Endosulphan, alpha	0.15
Endosulphan, beta	0.16
Endosulphan Sulphate	0.21
Endrin	0.17
Endrin Aldehyde	0.26
Endrin Ketone	0.06
HCH, alpha	0.24
HCH, beta	0.31
HCH, gamma	0.30
HCH, delta	0.22
Heptachlor	0.27
Heptachlor Epoxide	0.19
Hexachlorobutadiene	0.12
Nonachlor, trans-	0.05
Nonachlor, cis-	0.05
Methoxychlor	0.13
Mirex	0.11
Octachlorostyrene	0.08
Technical Toxaphene	13.53

Supplemental Table S4. Detection limits, number and percent of samples above the detection limit, and percent recovery for elements in tree swallow liver tissue from the Duluth, MN area between 2019 – 2021.

Element	Detection limit µg/g dry weight	Number detected	Percent detected	Percent Recovery
Aluminum	0.925	17	27	82.6
Arsenic	0.053	14	22	102.7
Barium	0.052	10	16	98.6
Beryllium	0.052	0	0	99.8
Boron	0.528	13	21	97.8
Cadmium	0.012	63	100	99.1
Chromium	0.052	4	6	84.3
Cobalt	0.021	52	83	99.2
Copper	0.245	63	100	96.3
Iron	0.405	63	100	110.6
Lead	0.052	10	16	100.0
Lithium	0.219	0	0	0
Magnesium	1.73	63	100	94.1
Manganese	0.081	63	100	104.6
Mercury	0.006	63	100	100.3
Molybdenum	0.149	63	100	107.1
Nickel	0.209	0	0	98.0
Selenium	0.098	63	100	100.5
Strontium	0.080	21	33	88.3
Thallium	0.012	16	25	98.9
Vanadium	0.052	0	0	100.0
Zinc	0.302	63	100	105.3

Suppl. Table S5. Geometric mean concentrations (ng/g wet wt., except as note below) and 95% confidence intervals (CIs) for other chemical contaminants in tree swallow nestling carcasses at sites in the Duluth, MN area in 2020 – 2021.

Contaminant	St. Louis River	North region				Reference
	Boy Scout Landing	ANG	Martin Road	Rice Lake North	UMD	Boulder Lake
	n = 6	n = 5	n = 5	n = 1	n = 1	n = 5
Total PCBs[1]	34.15 A[2] (23.94-48.72)	27.92 AB (5.48-142.11)	13.58 AB (8.71-21.17)	8.77 AB	10.77 AB	7.50 B (4.99-10.55)
PCB TEQs[3]	0.0051 A (0.0007-0.036)	0.001 AB (0.00003-0.39)	0.00005 AB (0.00003-0.00009)	0.00004 AB	0.00005 AB	0.00003 B (0.00002-0.00003)
Total PBDEs (pg/g)	3008[4] A (1930.8-4686.5)	1987 A (770.6-5123.9)	1841 A (1020.4-3320.4)	Not Analyzed	1314 A	Not Analyzed
p,p'-DDE	4.19 A (3.28-5.35)	4.95 A (3.01-8.16)	4.30 A (3.37-5.49)	4.21 A	6.89 A	5.79 A (4.72-7.10)
p,p'-DDD	0.08 AB (0.03-0.17)	0.41 A (0.09-1.91)	0.11 AB (0.07-0.18)	0.30 AB	0.03 B	0.18 AB (0.11-0.29)
Dieldrin	0.20 A (0.08-0.52)	0.09 A	0.13 A (0.04-0.37)	0.09 A	0.67 A	0.26 A (0.14-0.52)
Hexachlorobenzene	0.60 A (0.51-0.71)	0.33 BC (0.29-0.37)	0.33 BC (0.27-0.40)	0.45 AB	0.26C	0.45 AB (0.37-0.54)
Pentachlorobenzene	0.14 AB (0.08-0.25)	0.08 B	0.10 B (0.07-0.14)	0.08 B	0.41 A	0.12 B (0.09-0.15)
Trans-nonachlor	0.22 B (0.18-0.28)	0.25 B (0.16-0.39)	0.29 B (0.15-0.55)	0.39 AB	1.79 A	0.18 B (0.07-0.42)
Cis-nonachlor	0.06 AB (0.03-0.12)	0.04 B (0.02-0.07)	0.10 AB (0.06-0.19)	0.15 AB	0.26 A	0.07 AB (0.04-0.15)
Mirex	0.24 A (0.22-0.26)	0.38 A (0.11-1.32)	0.38 A (0.24-0.61)	0.20 A	0.41 A	0.21 A (0.14-0.33)
Total Pesticides	6.74 A (5.21-8.72)	7.82 A (4.75-12.87)	6.18 A (4.44-8.59)	5.70 A	13.42 A	9.16 A (8.24-10.17)

Note: polychlorinated biphenyls (PCBs), toxic equivalents (TEQ), polybrominated diphenyl ethers (PBDEs)

[1] Bonferroni alpha = 0.6 to force mean separations among sites
[2] Means sharing same letter are not signific ally different (1-way ANOVA) among sites

[3] Bonferroni alpha = 0.1 to force mean separation
[4] Sample size for PBDE analyses was 4

Supplemental Table S6. Geometric means (ng/g wet wt.), 95% confidence intervals (CIs), minimum and maximum values for contaminants detected in greater than 50% of tree swallow egg samples from Boulder Lake, north of Duluth, MN in 2020.

Contaminant	Boulder Lake<br><br>n = 2
Total PCBs	48.26<br>(1.46-1600.04)
PCB TEQs	0.04<br>(0.0000007-2297.82)
p,p,'DDE	54.20<br>(0.18-16240.69)
p,p'DDD	0.17<br>(0.0003-103.13)
Dieldrin	0.20<br>(0.000004-11006.21)
HCB	2.43<br>(1.30-4.51)
Pentachlorobenzene	0.17<br>(0.00003-1122.68)
Trans-nonachlor	1.15<br>(0.0000000001-11561142719)
Cis-nonachlor	0.66<br>(0.0000001-6212153.25)
Mirex	2.12<br>(2.06-2.19)
Total Pesticides	75.08<br>(3.54-1594.34)

Note: polychlorinated biphenyls (PCBs), toxic equivalents (TEQs)

Suppl. Table S7. Geometric mean concentrations (µg/g dry wt.), 95% confidence intervals (CIs), and minimum and maximum values for elements detected in >50% of tree swallow nestling liver samples collected in the Duluth, MN area, 2019-2021.

		Potentially toxic					Non-toxic				
Site	Sample size	Cadmium	Cobalt	Mercury	Molybdenum	Selenium	Copper	Iron	Magnesium	Manganese	Zinc
Reference											
Boulder Lake	7	0.03 B[1]<br>0.03-0.04<br>(0.027-0.051)	0.03 AB[2]<br>0.02-0.05<br>(1ND[3]-0.057)	0.16 AB<br>0.13-0.19<br>(0.097-0.192)	2.02 ABC<br>1.92-2.11<br>(1.91-2.2)	2.14 DE<br>1.87-2.45<br>(1.8-2.74)	15.59 A<br>10.3-23.7<br>(12.4-43.4)	1423 AB[2]<br>1147-1764<br>(1020-2150)	736 AB[2]<br>697-778<br>(693-821)	4.56 B<br>4.13-5.05<br>(3.7-5.21)	74.5 B<br>71.4-77.9<br>(69.4-78.8)
North region											
Rice Lake North	1	0.039 AB	0.026 AB	0.10 BC	2.11 ABC	2.5 CDE	35.10 A	1290 AB	763 AB	6.34 A	72.6 B
Martin Road		0.04 B<br>0.03-0.05<br>(0.025-0.049)	0.03 AB<br>0.02-0.04<br>(0.022-0.039)	0.10 BC<br>0.08-0.12<br>(0.077-0.125)	1.91 ABC<br>1.68-2.16<br>(1.61-2.07)	1.92 E<br>1.72-2.15<br>(1.66-2.07)	18.05 A<br>14.5-22.5<br>(14.4-22.9)	1394 AB<br>875-2220<br>(817-2030)	771 AB<br>740-803<br>(743-792)	4.86 AB<br>3.97-5.96<br>(3.86-5.88)	82.4 AB<br>70.9-95.6<br>(67.3-91.9)
ANG	5	0.07 A<br>0.05-0.10<br>(0.054-0.102)	0.02 AB<br>0.01-0.04<br>(1ND-0.029)	0.07 C<br>0.05-0.12<br>(0.055-0.134)	1.73 C<br>1.31-2.27<br>(1.27-2.32)	1.88 E<br>1.62-2.18<br>(1.68-2.2)	16.70 A<br>12.4-22.5<br>(12.1-22.1)	740.0 B<br>620-884<br>(634-922)	731 B<br>676-791<br>(683-786)	5.81 AB<br>4.81-7.02<br>(4.7-7.22)	71.2 B<br>59.7-84.8<br>(59.2-83.7)
St. Louis River											
Thomson Reservoir	5	0.04 AB<br>0.03-0.05<br>(0.032-0.050)	0.06 A<br>0.04-0.09<br>(0.040-0.088)	0.14 AB<br>0.117-0.167<br>(0.111-0.164)	2.48 A<br>2.08-2.95<br>(1.99-2.78)	4.54 A<br>3.33-6.17<br>(3.25-6.13)	19.37 A<br>15.8-23.8<br>(15.1-23.1)	1452 A<br>745-2831<br>(669-2330)	813 A<br>775-853<br>(764-840)	4.63 AB<br>4.28-5.01<br>(4.22-4.98)	102.3 A<br>92.1-113.8<br>(95.2-117)
Boy Scout Landing	6	0.05 AB<br>0.04-0.07<br>(0.030-0.073)	0.02 B<br>0.01-0.04<br>(2ND-0.041)	0.18 A<br>0.13-0.24<br>(0.121-0.272)	1.81 BC<br>1.54-2.14<br>(1.43-2.19)	2.96 BCD<br>2.52-3.48<br>(2.42-3.72)	15.95 A<br>14.3-17.8<br>(13.9-17.6)	1188 AB<br>932-1515<br>(830-1520)	764 AB<br>705-827<br>(678-812)	5.07 AB<br>4.42-5.81<br>(3.96-5.68)	75.5 B<br>66.6-85.6<br>(64.7-86.9)

Munger Landing	10	0.04 B<br>0.03-0.04<br>(0.026-0.047)	0.02 AB<br>0.02-0.03<br>(2ND-0.034)	0.17 ABC<br>0.15-0.19<br>(0.12-0.245)	2.15 ABC<br>2.05-2.26<br>(1.86-2.33)	3.43 ABC<br>3.16-3.72<br>(2.84-4.14)	23.94 A<br>18.6-30.8<br>(15.8-49.6)	1075 AB<br>896-1289<br>(685-1370)	796 AB<br>759-834<br>(717-884)	5.16 AB<br>4.67-5.71<br>(4.3-6.03)	80.2 AB<br>70.2-91.8<br>(59.4-97.6)
Stryker Bay	5	0.05 AB<br>0.04-0.07<br>(0.038-0.076)	0.03 AB<br>0.03-0.05<br>(0.029-0.052)	0.135 AB<br>0.110-0.166<br>(0.112-0.165)	2.22 ABC<br>1.92-2.56<br>(1.89-2.61)	3.61 AB<br>3.38-3.85<br>(3.37-3.81)	18.17 A<br>11.4-29.0<br>(14-35)	937 AB<br>651-1350<br>(682-1430)	789 AB<br>754-826<br>(759-824)	5.0 AB<br>4.59-5.45<br>(4.65-5.45)	90.1 AB<br>79.9-101.5<br>(79.8-104)
Erie Ponds	13	0.04 AB<br>0.03-0.05<br>(0.027-0.073)	0.02 AB<br>0.01-0.04<br>(4ND-0.249)	0.14 AB<br>0.12-0.16<br>(0.103-0.199)	2.15 ABC<br>2.00-2.31<br>(1.71-2.53)	2.56 CDE<br>2.37-2.76<br>(2.06-3.21)	18.60 A<br>16.3-21.3<br>(13.5-27.5)	1242 AB<br>1041-1481<br>(704-2140)	749 AB<br>719-780<br>(685-848)	4.95 AB<br>4.69-5.22<br>(4.26-5.76)	78.4 AB<br>73.6-83.4<br>(66.7-91.1)
Miller Creek	6	0.05 AB<br>0.04-0.07<br>(0.033-0.069)	0.02 AB<br>0.01-0.04<br>(1ND-0.027)	0.07 C<br>0.06-0.08<br>(0.057-0.183)	2.40 AB<br>2.25-2.57<br>(2.25-2.54)	2.22 DE<br>2.01-2.44<br>(2.05-3.67)	20.84 A<br>15.0-29.0<br>(14.7-28.3)	1300 AB<br>1099-1538<br>(1100-1750)	776 AB<br>761-791<br>(762-895)	5.64 AB<br>4.31-7.38<br>(4.07-7.45)	67.8 B<br>63.9-72.0<br>(64.5-109)
P-value[4]		0.0003	0.0372	<0.0001	<0.0001	<0.0001	0.0534	0.0001	0.0438	0.0335	0.0002

[1] Means sharing same letter are not significantly different among sites.
[2] Bonferroni means only separated when P set at 0.25 (Co), 0.1 (Fe), and 0.7 (Mg).
[3] ND = number below the detection limit.
[4] P-value for 1-way ANOVA.

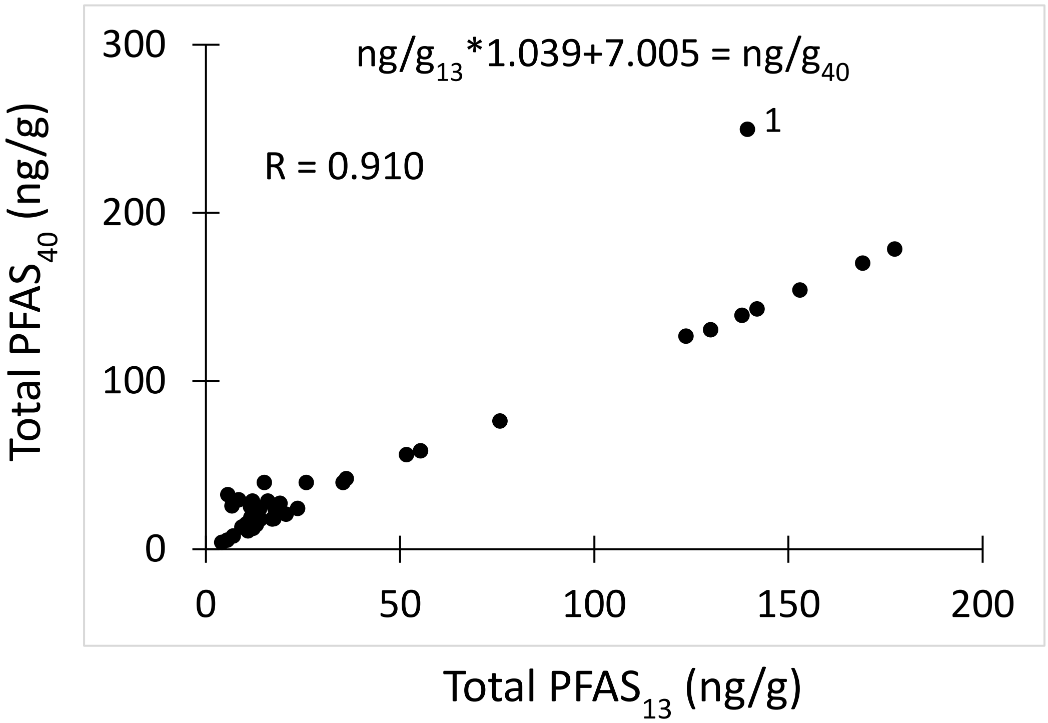

[1] Site is ANG

Figure S1: Correlation between original 13 per- and polyfhuoroalkyl substances ($PFAS_{13}$) and current 40 PFAS ($PFAS_{40}$) in tree swallow nestlings from the Duluth, MN area in 2021.

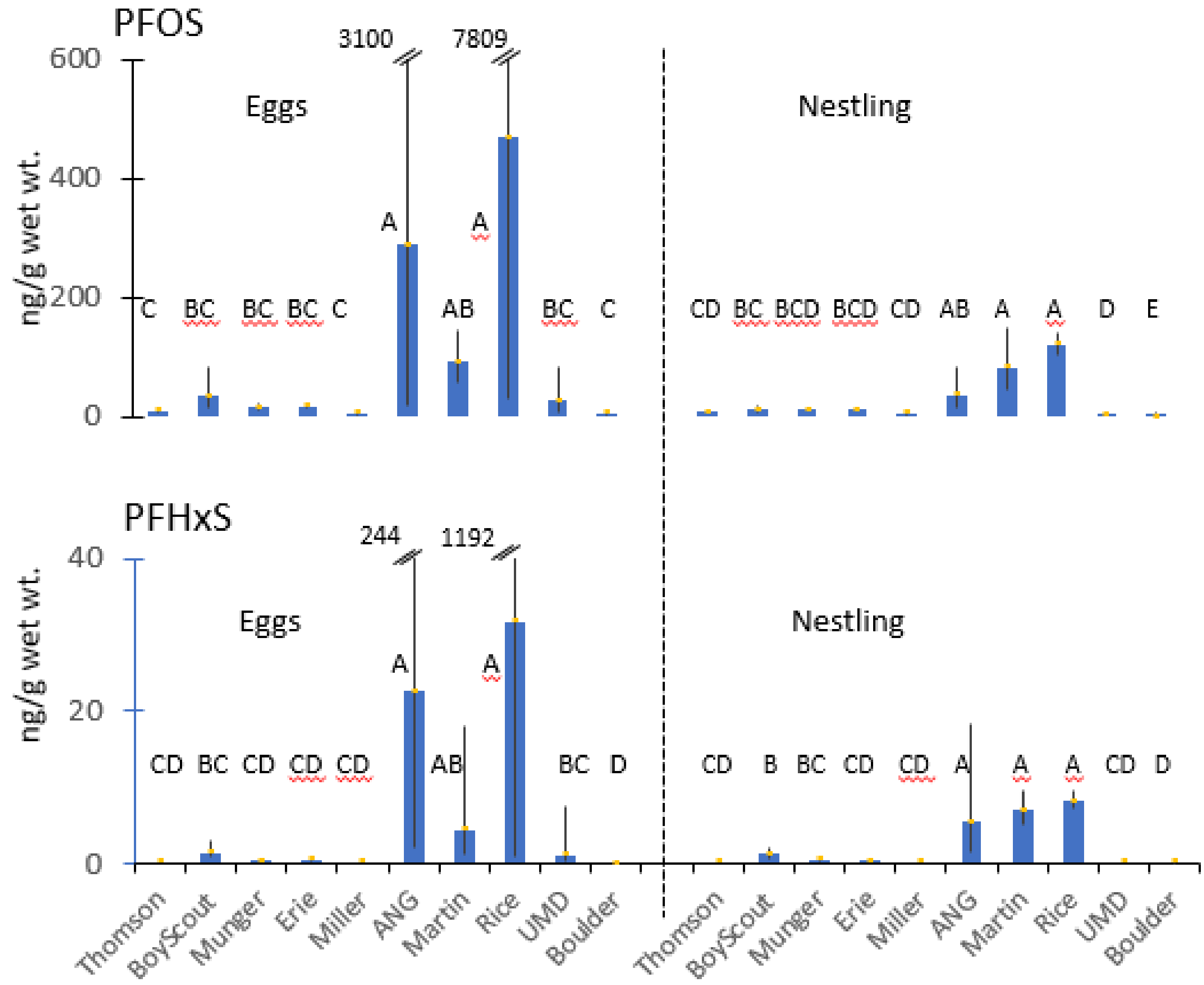

Figure S2: Geometric means (bars, ng/g wet wt.) and 95% confidence intervals (vertical lines) for perfluorooctane sulfonate (PFOS, upper panel) and perfluorohexane sulfonate (PFHxS, lower panel) in tree swallow eggs (left side) and nestling carcasses (right side) from the Duluth, MN area, 2019 - 2021. Means sharing same letter are not significantly different, 1-way ANOVA.

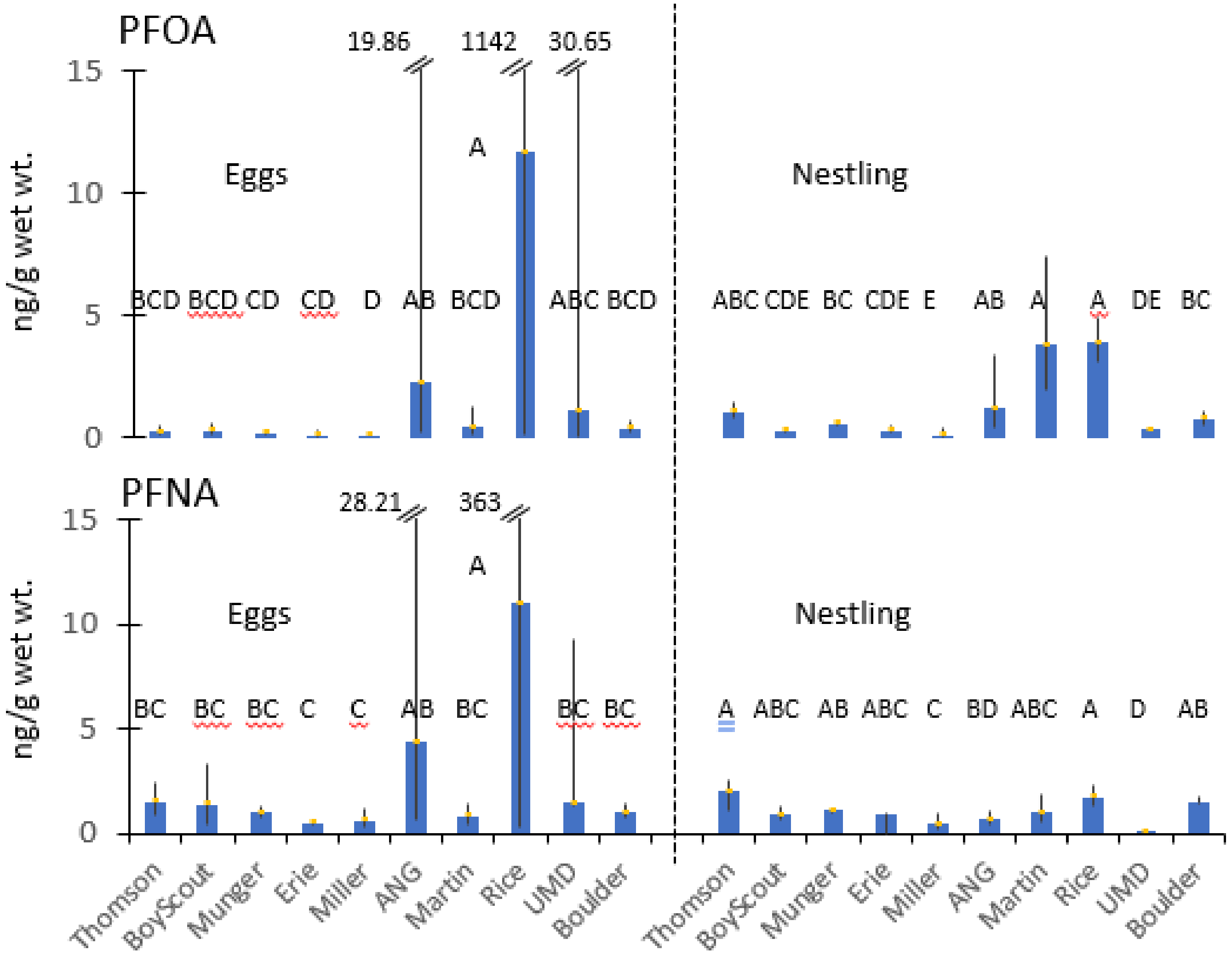

Figure S3: Geometric means (bars, ng/g wet wt.) and 95% confidence intervals (vertical lines) for perfluorooctanoate (PFOA, upper panel) and perfluorononanoate (PFNA, lower panel) in tree swallow eggs (left side) and nestling carcasses (right side) from the Duluth, MN area, 2019 - 2021. Means sharing same letter are not significantly different, 1-way ANOVA.

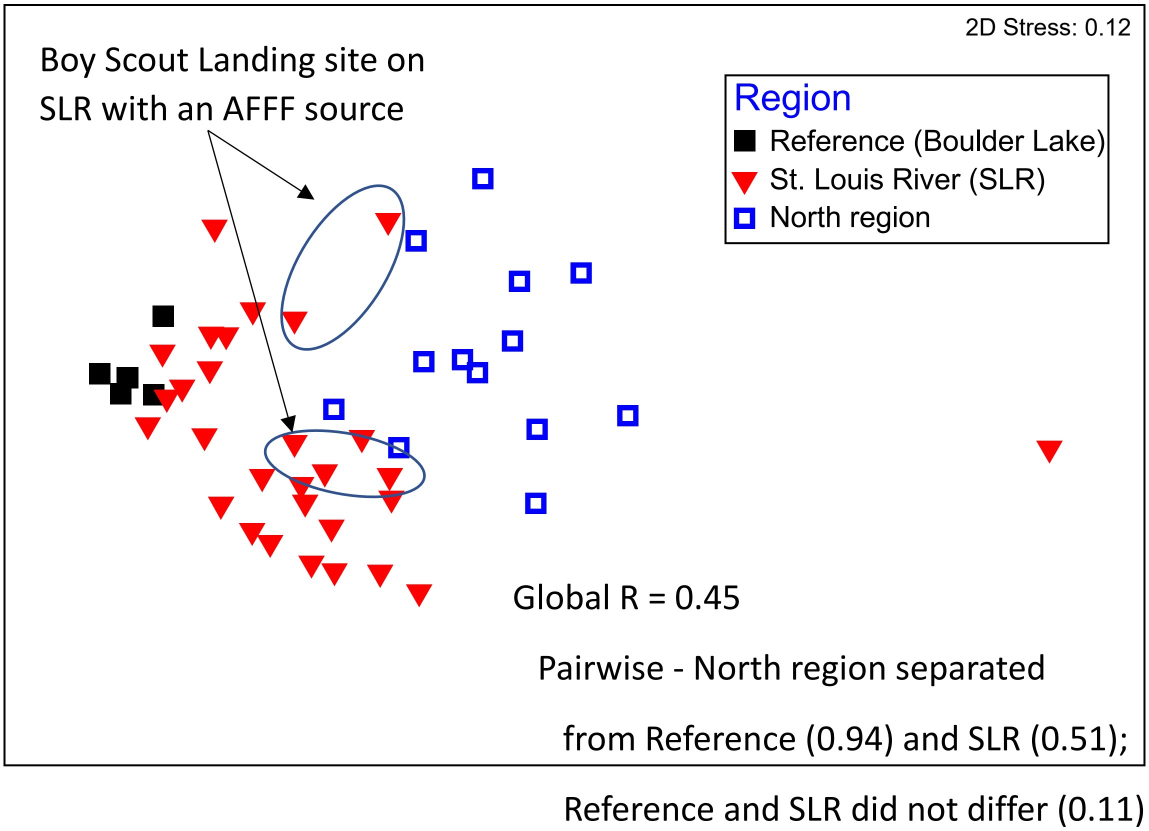

Figure S4: Nonmetric multidimensional scaling (NMDS) plot of PFAS in tree swallow egg samples (2020 & 2021) in the Duluth, MN area separated by region for n = 21 PFAS detected in >2 samples. Note that NMDS plots are unitless.

Note: AFFF = aqueous film forming foams

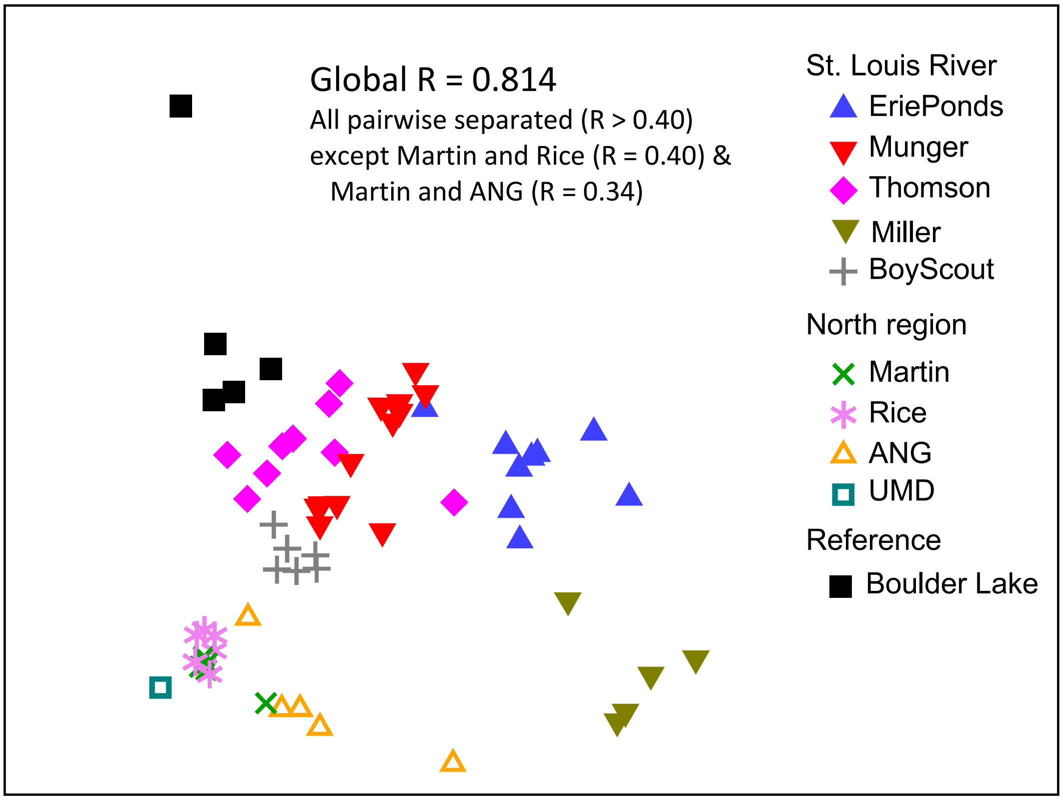

Figure S5: Nonmetric multidimensional scaling (NMDS) plot of PFAS by individual site in tree swallow nestling samples (2010 & 2021) in the Duluth, MN area for n = 25 PFAS detected in >2 samples. Note that NMDS plots are unitless.

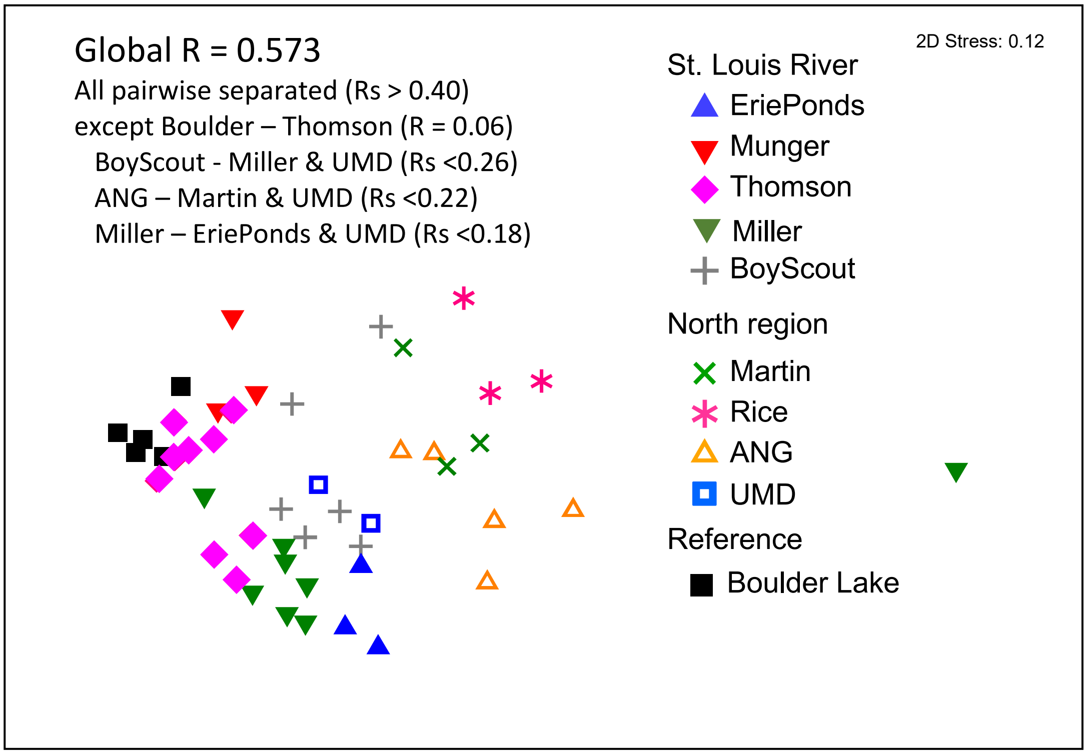

Figure S6: Nonmetric multidimensional scaling (NMDS) plot of PFAS by individual site in tree swallow egg samples (2010 & 2021) in the Duluth, MN area for n = 21 PFAS detected in >2 samples. Note that NMDS plots are unitless.
